# Structure and Distribution of Centromeric Retrotransposons at Diploid and Allotetraploid *Coffea* Centromeric and Pericentromeric Regions

**DOI:** 10.3389/fpls.2018.00175

**Published:** 2018-02-15

**Authors:** Renata de Castro Nunes, Simon Orozco-Arias, Dominique Crouzillat, Lukas A. Mueller, Suzy R. Strickler, Patrick Descombes, Coralie Fournier, Deborah Moine, Alexandre de Kochko, Priscila M. Yuyama, André L. L. Vanzela, Romain Guyot

**Affiliations:** ^1^Laboratory of Cytogenetics and Plant Diversity, Department of General Biology, Center for Biological Sciences, State University of Londrina, Londrina, Brazil; ^2^Department of Electronics and Automatization, Universidad Autónoma de Manizales, Colombia; ^3^Nestlé R&D Tours, Notre-Dame d'Oé, Tours, France; ^4^Boyce Thompson Institute, Cornell University, Ithaca, NY, United States; ^5^Nestlé Institute of Health Sciences, Lausanne, Switzerland; ^6^Institut de Recherche pour le Développement, UMR DIADE, EvoGec, Montpellier, France; ^7^Institut de Recherche pour le Développement, CIRAD, Univ. Montpellier, UMR IPME, Montpellier, France

**Keywords:** coffee, CRM lineages, FISH, *Gypsy*, pseudochromosomes, proximal chromosome regions, centromeres

## Abstract

Centromeric regions of plants are generally composed of large array of satellites from a specific lineage of *Gypsy* LTR-retrotransposons, called Centromeric Retrotransposons. Repeated sequences interact with a specific H3 histone, playing a crucial function on kinetochore formation. To study the structure and composition of centromeric regions in the genus *Coffea*, we annotated and classified Centromeric Retrotransposons sequences from the allotetraploid *C. arabica* genome and its two diploid ancestors: *Coffea canephora* and *C. eugenioides*. Ten distinct CRC (Centromeric Retrotransposons in *Coffea*) families were found. The sequence mapping and FISH experiments of CRC Reverse Transcriptase domains in *C. canephora, C. eugenioides*, and *C. arabica* clearly indicate a strong and specific targeting mainly onto proximal chromosome regions, which can be associated also with heterochromatin. PacBio genome sequence analyses of putative centromeric regions on *C. arabica* and *C. canephora* chromosomes showed an exceptional density of one family of CRC elements, and the complete absence of satellite arrays, contrasting with usual structure of plant centromeres. Altogether, our data suggest a specific centromere organization in *Coffea*, contrasting with other plant genomes.

## Introduction

LTR-retrotransposons pertain to the Class I of Transposable Elements (TEs), they move via the synthesis of an intermediate RNA using “copy and paste” mechanisms (Wicker et al., 2007). Due to their mobility, LTR-retrotransposons are the most abundant TEs (Grandbastien, 2015). They contribute to the variation of genome size and structure observed in plants (Piegu et al., 2006; Heslop-Harrison and Schwarzacher, 2011; Tenaillon et al., 2011).

LTR-retrotransposons are classified into *Copia* and *Gypsy* superfamilies according to their coding domain internal organization (Schnable et al., 2009; Gao et al., 2012; Bennetzen and Wang, 2014). Each *Copia* and *Gypsy* superfamily is sub-classified into lineages and families (Wicker et al., 2007), according to coding region similarities and overall structures (Llorens et al., 2009). For plant genomes, *Copia* is sub-classified into *Tork, Retrofit, Oryco, SIRE*, and *Bianca*, while *Gypsy* is sub-classified into *TAT, Athila, Galadriel, Reina, Del*, and *CRM* (Llorens et al., 2009, 2011), based on Reverse-Transcriptase (RT) domain phylogenetic analyses. *Gypsy* lineages are also grouped into different branches according to the presence of a chromodomain; grouping together *Galadriel, Reina, Del*, and *CRM* lineages into the Chromovirus branch.

*Copia* and *Gypsy* superfamilies can be found distributed in blocks or dispersed along plant chromosomes (Lopes et al., 2013; Santos et al., 2015; Zhang et al., 2017). One notable exception is the Centromeric Retrotransposon lineage of Chromovirus (*CRM* or Centromeric Retrotransposon of Maize), which appears located preferentially into proximal chromosome regions or “centromeric regions” (Nagaki et al., 2005; Bao et al., 2006; Liu et al., 2008; Du et al., 2010; Sharma and Presting, 2014). CRMs carry heterogeneous domains at the C-terminus of the integrase that may be linked to their chromosomal distribution. A chromodomain (CHRomatin Organization MOdifer domain) or a targeting domain called CR motif were identified (Houben et al., 2007; Neumann et al., 2011). These domains are probably able to interact with the CENH3 protein, suggesting that Centromeric Retrotransposons (CR) participate in centromere function. Plant centromeric regions can be composed of large arrays of CR elements inserted into specific satellite DNA (Cheng et al., 2002; Houben et al., 2007; Marques et al., 2015; Santos et al., 2015). Although relatively few centromeric regions have been studied in plants, especially due to difficulties to sequence and assemble regions with a high content of repetitive sequences, Neumann et al. (2011) separated CR elements into three groups according to their properties and chromosomal distribution: Group A carrying a CR motif and Group B lacking any targeting domain, both localized in centromeric regions; and Group C containing a chromodomain and dispersed along chromosomes.

The *Coffea* genus (Rubieaceae) comprises 125 species (Hamon et al., 2017). All species are diploids, except *Coffea arabica* (2*n* = 4*x* = 44), that arose from a recent hybridization between *C. canephora* and *C. eugenioides* (Lashermes et al., 1999; Yu et al., 2011). The recent sequencing of *C. canephora* genome revealed an important contribution of transposable elements (>50%). Most of them fell into the LTR-retrotransposons order (Denoeud et al., 2014). Several international sequencing initiatives are targeting the *C. arabica* genome using Pacific Biosciences (PacBio) single molecule sequencing (Mueller et al., 2015). This technique, allowing the sequencing of complex regions with a high content of repeated sequences, offers the opportunity to study the composition and organization of centromeric regions. In this study, we identified and compared 10 families of Centromeric Retrotransposons in the forthcoming PacBio genomes of *C. canephora, C. eugenioides*, and *C. arabica*. In situ hybridization using conserved RT probes showed CRCs located in proximal and interstitial chromosome regions. Finally, annotation and comparison of centromeric region rich in CRC elements revealed dynamic changes targeting LTR retrotransposons, but also the complete absence of tandem repeats usually associated with CRC elements.

## Materials and methods

### Genome sequencing

Genomic DNA was extracted from leaves using DNeasy Plant Maxi Qiagen Kit. For long read sequencing, 20 Kb libraries were prepared following Pacific Biosciences (PacBio) protocol and Blupippin size selection. Sequencing was performed on the PacBio RSII platform, and specifications are described in Supplemental data 1. For short read sequencing, libraries were prepared with the KAPA HyperPlus kits, following manufacturer recommendation and sequenced on Illumina HiSeq2500 using PE flow cells and V4 chemistry. Genomes were assembled using Falcon and Falcon unzip from Pacific Bioscience (https://github.com/PacificBiosciences/FALCON).

### *In silico* analyzes

Genomes of *C. canephora* (DH200-94-V.2)*, C. eugenioides* (BU-A) and *C. arabica* (accession Et39), were kindly provided by the ACGC (2014) with the single molecule real-time (SMRT, Pacific Biosciences—PacBio). The three genomes were sequenced using the long-read Pacific Bioscience technology (Mueller et al., 2015). *C. canephora* genome assembly was finished using both Bionano genome mapping and Dovetail Hi-C scaffolding technologies (ACGC, unpublished results).

### Transposable element annotations and analyses

Sequenced genomes served as source for searching and comparing LTR-retrotransposons using the LTR_STRUC (McCarthy and McDonald, 2003). Putative retrotransposons sequences were classified into *Gypsy* and *Copia* superfamilies according to their similarity against the Gypsy Database protein domains (http://www.gydb.org/index.php/Main_Page) as implemented in the *Impactor* program (Orozco et al. unpublished. Available upon request). Putative reverse transcriptase (RT) domain from the *Gypsy* superfamilies were identified using BLASTX (Altschul et al., 1997) and extracted and translated into amino acids using Genewise (Birney et al., 2004) with a minimum length of 150 residues as in Guyot et al. (2016). For each coffee genome, RT domains from *Gypsy* LTR-RTs were aligned using MUSCLE (Edgar, 2004) with RT reference domains from the Gypsy Database. Aligned sequences were used to construct a bootstrapped neighbor joining phylogenetic tree (1,000 bootstrap) with ClustalW (Thompson et al., 1994), edited using FigTree (http://tree.bio.ed.ac.uk/software/figtree/).

The coffee sequences from the *CRM* lineage and called hereafter CRC (Centromeric Retrotransposons of *Coffea*) sequences were identified from the NJ tree. These sequences were sub-classified into groups according to tree conformation and bootstrap values. Groups were validated by alignments using dotter (Sonnhammer and Durbin, 1995), stretcher (EMBOSS) and plotcon (EMBOSS). LTR sequences with 99% identity based on LTR_STRUC were annotated using Artemis (Rutherford et al., 2000). Complete (i.e., a LTR-retrotransposon containing both LTR domains) and putative autonomous (i.e., a LTR-retrotransposon containing all coding domain involved in its mobility) elements were compared and grouped with the Mauve tool (http://darlinglab.org/mauve/mauve.html). Non-autonomous elements were classified into TRIM, LARD, and TR-GAG according to their length and domains as in Chaparro et al. (2015) and implemented in the *Impactor* program (Orozco et al., unpublished). A representative element of each group was submitted to GenBank under the following accession: A MG242426; B MG242427; C MG242428; D MG242429; E MG242430; F MG242431; G MG242432; H MG242433; Y MG242434; X MG242435.

### *In silico* estimation of CRC elements copy number and distribution

Assessment of the CRC elements copy number in *C. canephora, C. arabica*, and *C. eugenioides* PacBio sequences was done as in Dupeyron et al. (2017). Briefly, each representative copy of CRC groups was used for similarity searches against genomes using Censor (http://www.girinst.org/downloads/software/censor/). Copies are sorted according to their completeness and percentage of similarity when compared to the representative copy. Insertion times of selected LTR-RT were estimated as proposed by SanMiguel et al. (1998) and Guyot et al. (2016), with a substitution rate of 1.3 × 10^−8^, established by Ma and Bennetzen (2004). The distribution of RT domains was carried out using RepeatMasker (–div 20 option) while the distribution of complete elements, LTR and non-autonomous elements was performed using Censor with a minimum of 80% of nucleotides identity and 80% of sequence coverage.

The centromeric regions annotation was performed using RepeatMasker (–div 20 option) and edited with Artemis, and transposable elements density along genomic sequences was carried out using DensityMap (Guizard et al., 2016).

### Plant materials, DNA extraction, and probes production

Seedlings of *C. arabica, C. canephora*, and *C. eugenioides* were obtained from the Agronomic Institute of Paraná (IAPAR), Londrina, Paraná, Brazil, cultivated in pots in the green house of the Laboratory of Cytogenetics and Plant Diversity, State University of Londrina, Brazil. DNA extraction was performed as described by Romano and Brasileiro (1999). Quickly, young leaves were collected, macerated in liquid nitrogen and treated with CTAB extraction buffer. DNA was purified with phenol:chloroform (1:1, v:v) and chloroform:isoamyl alcohol (24:1, v:v) and precipitated in absolute ethanol. DNA concentration was estimated using a NanoDrop 2000 Spectrophotometer (Thermo Scientific). Primers were designed using OligoPerfect™Designer (http://tools.lifetechnologies.com). A conserved region located in the predicted Reverse Transcriptase (RT) coding region of each CRC group was amplified by PCR using a pair of RT primers (Forward: 5′ACTGTCGGGCTGTAAATGCT; Reverse: 5′CTGCGAACTCACGACATAGC). Reactions were done using *C. arabica, C. canephora*, and *C. eugenioides* genomic DNA as template, in a mix composed by 0.5 μL Taq Polymerase (5 U/μL), 2.5 μL 10 × buffer, 2.5 μL MgCl_2_ (50 mM), 1 μL of dNTP (10 mM), 1 μL of each primer at 10 mM and H_2_O, in a final volume of 25 μL. Reactions were checked with 1% agarose gel electrophoresis. Probes were obtained by PCR, using the product of a first PCR as template, in a new reaction containing dGTP (25%), dCTP (25%), dTTP (25%), dATP (17.5%), and Cy3-dUTP (7.5%).

### Cytogenetic analyses

Mitotic chromosomes were obtained from root tips treated with a saturated solution of paradichlorobenzene (PDB) for 1 h at room temperature plus 23 h at 14°C. Samples were fixed in a fresh solution of methanol: acetic acid (3:1, v:v) for 24 h, and stored at −20°C, or used immediately. Root-tips were softened in 2% cellulase plus 20% pectinase (v:v), both Sigma, at 37°C for 5 h, and squashed in a drop of 60% acetic acid. The cover slips were removed after freezing in liquid nitrogen, slides were air dried and used in FISH or C-CMA/DAPI banding procedures.

For FISH, a mixture of 30 μL containing 100% formamide (15 μL), 50% polyethylene glycol (6 μL), 20× SSC (3 μL), 100 ng calf thymus DNA (1 μL), 10% SDS (1 μL), and 100 ng probes (4 μL), was treated at 70°C for 10 min, placed on ice and immediately applied to the samples. Denaturation/hybridization was performed at 95, 50, and 38°C, 10 min each, followed by 37°C overnight in a humidified chamber. Post-hybridization washes were carried out in SSC buffer with about 70% stringency, mounted in 23 μL antifade solution (90% glycerol, 2.3% DABCO, 2% 20 mM Tris–HCl, pH 8.0, plus 1 μL of 2 μg/mL DAPI, and 1 μL of 2.5 mM MgCl_2_).

Chromosome banding was done using 3 days aged slides incubated in a solution of 45% acetic acid, 5% barium hydroxide, and 2× SSC, pH 7.0 (Schwarzacher et al., 1980, with modifications). Samples were stained with 0.5 mg/mL CMA_3_ for 1.5 h and 2 mg/mL DAPI for 30 min, and finally stained with a medium composed of glycerol/McIlvaine buffer (pH 7.0) 1:1 plus 2.5 mM MgCl_2_. FISH and C-CMA/DAPI chromosome images were acquired in gray-scale mode using a Leica DM4500B microscope, equipped with a Leica DFC300FX camera, and overlapped with blue for DAPI, greenish-yellow for CMA and red for Cy3, and processed using the Leica LAS software. Images were optimized for contrast and brightness using the GIMP 2.8 Image Editor.

## Results

### The *Gypsy* superfamily and the *CRM* lineage in coffee genomes

The search for complete LTR-retrotransposons sequences in *C. canephora, C. eugenioides* and *C. arabica* allowed to recognize 7,195, 3,590, and 3,877 elements, respectively. These were predicted and classified into 1,021 *Copia* and 2,222 *Gypsy* (*C. canephora*), 668 *Copia* and 950 *Gypsy* (*C. eugenioides*) and 743 *Copia* and 1226 *Gypsy* (*C. arabica*). The remaining predicted elements were identified into non-autonomous LTR-retrotransposons or into unclassified autonomous elements according to similarities to GAG-POL regions available at the Gypsy Database. For the *Gypsy* superfamily, the LTR-retrotransposon lineages (*Del, Galadriel, Reina, CRM, Athila*, and *TAT*), were found in the three *Coffea* genomes, using a BLAST based analysis and a RT based phylogenetic analysis (Supplemental datas 2, 3), and the *CRM* lineage was particularly analyzed.

The *CRM* lineage represented 499, 223, and 262 of complete annotated elements in *C. canephora, C. eugenioides, and C. arabica* genomes, respectively. Manual inspection revealed that 367 (73.55%), 124 (55.61%), and 113 (43.13%) elements were found complete for *C. canephora, C. eugenioides, and C. arabica*, respectively, since no large deletion affected these sequences. The RT amino acid sequences of the *CRM* lineage from the three coffee species were grouped together, aligned and displayed with a N.J. phylogenetic tree (Supplemental data 4). Ten phylogenetic groups were defined according to the structure and similarity of these domains (Table 1 and Supplemental data 5). The Centromeric Retrotransposons of *Coffea* were grouped and named here as follow A, B, C, E, D, F, G, H, X, and Y.

**Table 1:** Matrix of RT domain identity between CRC groups in *Coffea eugenioides, C. canephora*, and *C. arabica*.

	**CR Groups**								
	***C. canephora***	**A (%)**	**B (%)**	**C (%)**	**D (%)**	**E (%)**	**F (%)**	**G (%)**	**H (%)**
*C.eugenioides*	X	48	57	61	57	60	61	64	56
	A	88	49	48	44	48	47	47	45
	B	48	91	58	54	57	58	58	55
	C	45	56	82	57	59	58	59	57
	E	49	57	60	59	92	61	61	57
	F	47	57	60	59	61	93	61	59
	H	46	55	58	57	58	60	59	80
	***C. arabica***	**X (%)**	**Y (%)**	**B (%)**	**C (%)**	**E (%)**	**F (%)**	**G (%)**	**H (%)**
*C.eugenioides*	X	87	59	58	60	60	60	64	57
	A	46	47	48	47	47	47	47	44
	B	57	57	97	57	57	57	58	54
	C	59	59	56	84	60	57	60	57
	E	59	60	57	60	93	61	61	57
	F	60	59	58	60	61	90	61	59
	H	58	56	55	57	58	59	59	80
	***C. arabica***	**Y (%)**	**X (%)**	**B (%)**	**C (%)**	**E (%)**	**F (%)**	**G (%)**	**H (%)**
*C. canephora*	A	47	47	49	48	48	48	47	45
	B	56	57	91	58	58	57	58	54
	C	61	59	58	93	60	60	60	56
	D	57	58	54	56	60	57	58	57
	E	60	60	58	60	97	61	61	57
	F	59	60	58	59	61	94	61	59
	G	60	63	58	60	61	60	98	58
	H	56	57	55	56	57	58	58	92

*The letters A, B, C, D, E, F, G, H, X, and Y correspond to CRC groups, as defined by the phylogenetic analysis. Values highlighted in gray represent the highest percentage of identity observed between groups*.

**Figure 1:**
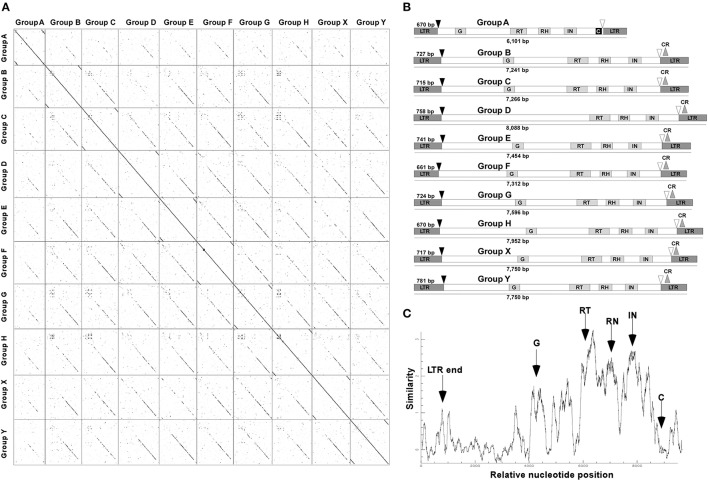
Structure and conservation of the *Gypsy* CRC LTR-retrotransposons in *Coffea arabica, C. canephora* and *C. eugenioides*. **(A)** Dotter alignments between the 10 groups of CRC found by LTR_STRUC against themselves. **(B)** Structural features of CRC groups. LTR, Long Terminal Repeats; G, GAG domain; RT, Reverse Transcriptase; RH, RNAse H; IN, Integrase; C, Chromodomain; CR, CR motif. The dark arrows indicate the PBS sites while the white arrows indicate the PPT sites. **(C)** Nucleotide similarity plot with the 10 groups of CRC. The positions of the different domains are indicated.

About 60 CRC sequences per genome, from the different groups, showing >99% of nucleotide identity between both LTR of the same element were carefully annotated and compared (Figure 1). Only elements from the A group presented a chromodomain, with zinc finger/HHCC motif at their C-terminus downstream the INT region, while elements from other groups exhibited a CR motif (Figure 1B) at their C-terminal regions, and a poly-A motif upstream the GAG region (data not shown). Autonomous elements of each group showed a variable length from 5,971 bp (Group_A) to 8,088 bp (Group_D), and a LTR size from 661 bp (Group_F) to 781 bp (Group_Y).

The alignment of complete elements into a matrix of nucleotides comparison showed discontinuous lines between groups, suggesting interrupted conservation along the different CRCs (Figure 1A). This discontinuous similarity was also confirmed with a nucleotide similarity plot of the full-length sequences of the 10 CRC groups (Figure 1C). The RT domain comparison at the nucleotide level showed a high conservation among elements within each group, independent of the species they are issued (from 80 to 98%), and a distant conservation between elements of different groups, i.e., from 45 to 64% (Table 1). These results suggest that CRCs are distributed among different families in the *Coffea* genus.

### Non-autonomous CRC elements in *Coffea*

Non-autonomous CRC elements, lacking any coding regions as seen in Terminal Repeat in Miniature (TRIMs) or Large Retrotransposon Derivative (LARDs), or lacking the POL polyprotein region as in TR-GAGs, were also identified (Chaparro et al., 2015). CRC group alignments (80% identity cutoff) against the putative non-autonomous elements exhibited different structures, such as TRIMs (only in *C. canephora*), LARDs and TR-GAGs. The counting showed 268, 216 and 216 putative non-autonomous CRC for *C. canephora, C. eugenioides*, and *C. arabica*, respectively (Supplemental data 6). Among them, the group B (mainly TR-GAG elements), the H (mainly LARD elements) and the group C, showed the highest number of copies, whatever the genome analyzed. Only the chromodomain of group A did not show similarity to any non-autonomous element.

### *In silico* copy number estimation and insertion time of 10 CRC families

A total copy number of 359, 278, and 473 CRC elements (with >80% of both coverage and identity) were found in *C. canephora, C. eugenioides*, and *C. arabica*, respectively. Besides conserved copies, fragmented copies (with >10% of coverage and >80% of identity) represented 2,055, 2,064, and 3,478 CRC elements in *C. canephora, C. eugenioides*, and *C. arabica*, respectively (Table 2). For the three species, elements from the groups H and B outnumbered the other groups for complete (80-80) and fragmented copies (80-10). The allotetraploid genome of *C. arabica* contains, as expected, the highest copy number when compared to the diploid genomes of *C. canephora* and *C. eugenioides*.

**Table 2:** Estimation of the copy numbers of CRC elements in the *Coffea canephora, C. eugenioides*, and *C. arabica* genome sequences.

	***C.canephora* copies (80–80)**	***C.canephora* partial copies (80–10)**	***C.eugenioides* copies (80–80)**	***C.eugenioides* partial copies (80–10)**	***C.arabica* copies (80–80)**	***C.arabica* partial copies (80–10)**
Group A	8	85	7	103	13	156
Group B	81	841	66	705	121	1,149
Group C	18	86	16	202	28	303
Group D	6	63	19	96	18	164
Group E	49	259	39	188	50	476
Group F	60	144	29	148	63	265
Group G	47	90	3	55	20	153
Group H	84	412	88	460	142	674
Group X	1	13	4	0	7	17
Group Y	5	62	7	107	11	121
Total	359	2055	278	2,064	473	3,478

The nucleotide divergence and relative insertion time of complete CRC copies suggest a relatively recent insertion, or a high conservation of the whole sequences with a similar pattern in *C. arabica, C. eugenioides*, and *C. canephora* (Supplemental data 7A). For each CRC group, three peaks of copy number accumulation were observed for the H group in *C. canephora, C. eugenioides* and *C. arabica*, while for the C group four and two peaks of copy number accumulation were noted for *C. eugenioides*, and for *C. canephora* and *C. arabica* (Supplemental datas 7B–D). Other and successive small peaks of copy number accumulation were observed for the E group, for example. This result suggested that the insertions of CRC are relatively recent, but that ancient activities may be detected, particularly for the group H.

**Figure 2:**
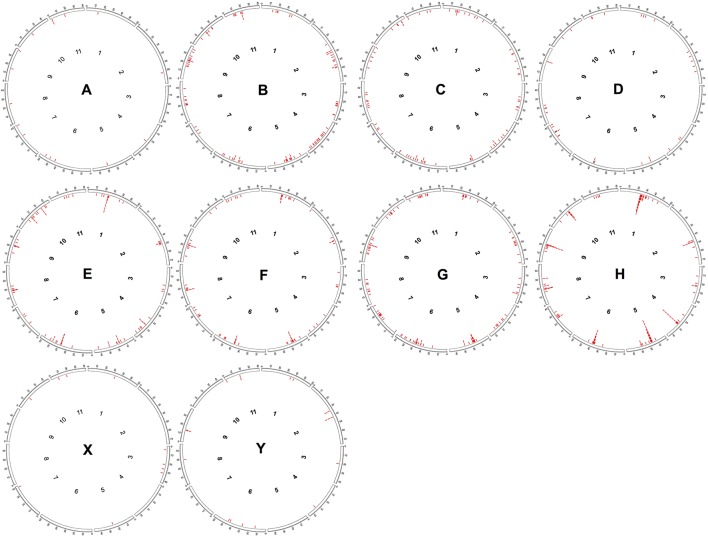
*In silico* distribution of RT domains from CRC groups along PacBio assembled pseudochromosomes of *Coffea canephora*. Each circle represents the distribution of one CRC group. Red lines represent the position of RT domains as found by RepeatMasker.

The distribution of CRC RT sequences along the *C. canephora* pseudochromosomes (Figure 2) showed that for some of them there is a clear accumulation of RT sequences in the central regions (pseudochromosomes 1, 2, 4, 5, 6, 8, 9, and 10). For the others, RT sequences were less concentrated, exhibiting a dispersed pattern, such as in the pseudochromosomes 3, 7 and 11. When we compare the distribution of these sequences of each CRC group along pseudochromosomes, it is possible to note that only the groups E and H showed a clear accumulation into median regions (Figure 2).

### Cytogenetic analysis

**Figure 3:**
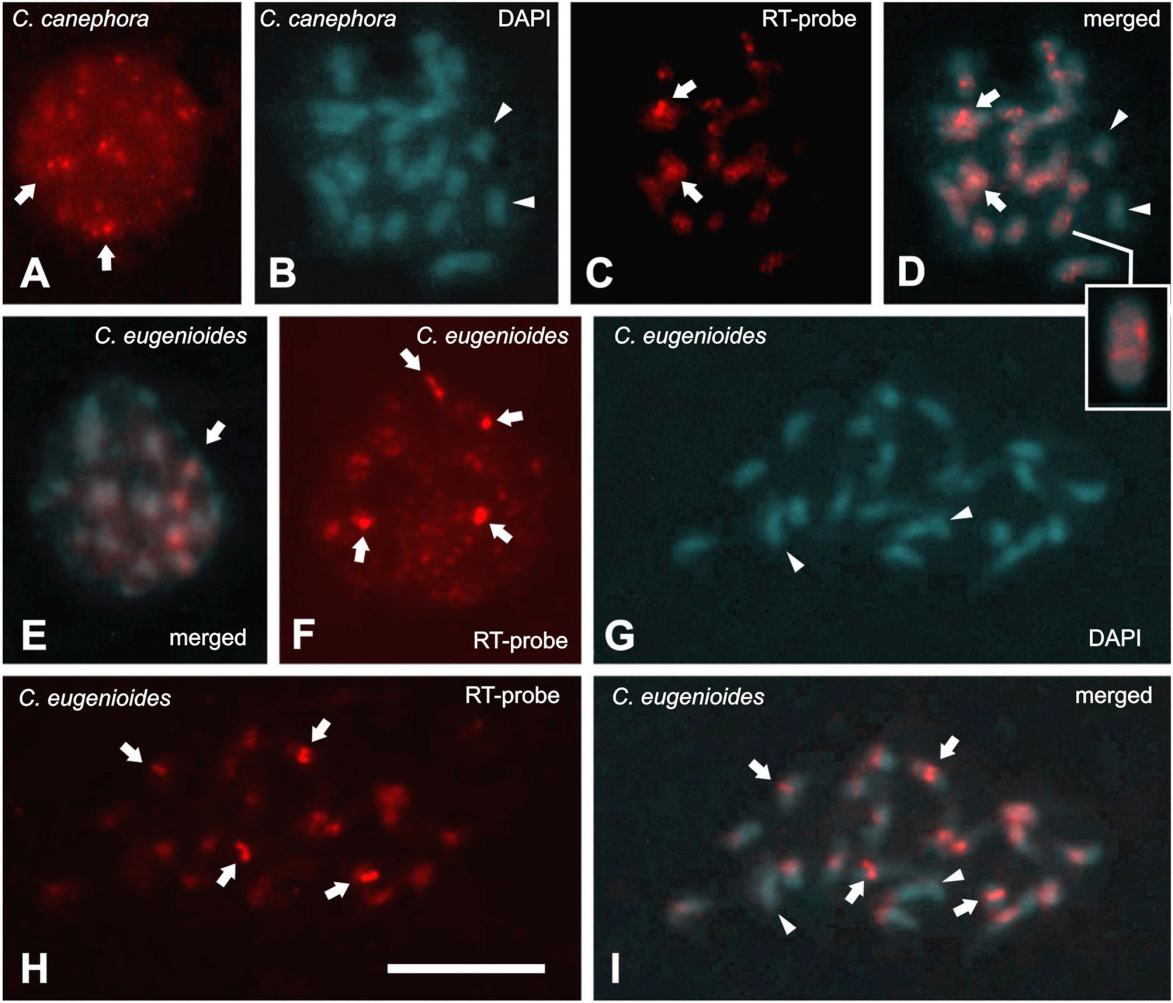
Fluorescence *in situ* hybridization (FISH) in nucleus and metaphases stained with DAPI (blue) and RT-CRC probe hybridized with Cy3-dUTP (red) in *Coffea canephora* **(A–D)** and *C. eugenioides* **(E–I)**. **(A)** Nucleus with scattered signals and two brighter signals (arrows). Metaphase stained with DAPI **(B)**, showing RT-CRC FISH signals **(C,D)** in the centromeres, proximal regions, including few chromosomes with scattered signals and proximal/interstitial dots (box), in red acquired and merged images. **(E)** Undifferentiated nucleus of *C. eugenioides* (Cy3/DAPI merged), showing scattered signals and four brighter signals Rabl-like organized, that are typical of centromeric location. Scattered and four large signals can also be observed in the red stained unpolarized nucleus **(F)**. Arrows point out the large FISH signals. **(G–I)** Prometaphase stained with DAPI and hybridized with RT-CRC probe. FISH indicates a predominance of centromeric-pericentromeric signals, including the four large signals detected in the nuclei (arrows). Arrowheads in **B, D, G**, and **I** indicate chromosomes without hybridization signals. Bar = 10 μm.

**Figure 4:**
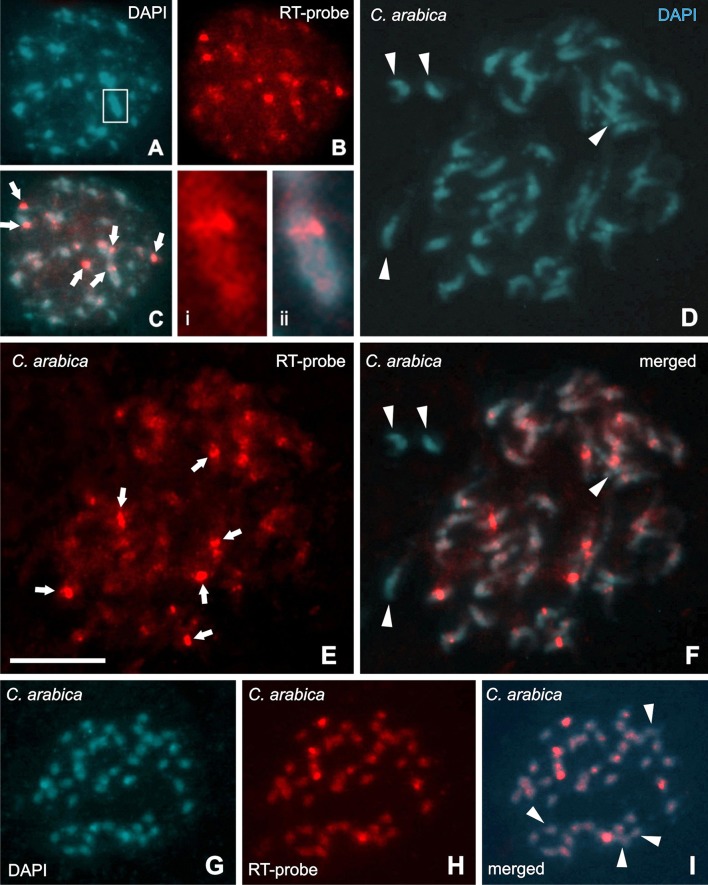
Fluorescence *in situ* hybridization (FISH) in nucleus, prometaphases and metaphases of *Coffea arabica*. Samples stained with DAPI appear in **(A,D)**, and with RT-CR FISH signals (red) are in the others. Nucleus showing scattered signals and with six brighter signals **(B)**, that are better observed in the merged image in **(C)** (arrows). Boxes **i** and **ii** (merged) show a well-defined RT-CRC signal into regions with more condensed chromatin. Prometaphases and metaphases hybridized with the RT-CRC probe **(E–I)** showing scattered signals, but with predominance of concentrated signals in the centromeric-pericentromeric regions (arrows in **E**) Arrowheads in **(D,F,I)** indicate chromosomes without hybridization signals. Bar = 10 μm.

FISH using a probe for RT conserved region, common for all CRC groups (Supplemental data 8), showed signals with differences in sizes and brightness on *C. arabica, C. canephora*, and *C. eugenioides* nuclei. Signals were distributed in all regions of differentiated cell nuclei (Figures 3A,F, 4A–C), and in a Rabl-like organization in undifferentiated cells (Figure 3E). Brighter signals appeared located in the proximal chromosome regions (see Table 3), but with variations within and between karyotypes of diploid species *C. canephora* with two signals (Figures 3B–D) and *C. eugenioides* with four signals (Figures 3G–I), and with six signals in the allotetraploid *C. arabica* (Figures 4D–I). In addition to the predominant signals into proximal regions, few chromosomes displayed scattered signals in proximal/interstitial dots (except for *C. eugenioides*). This is probably due to a smaller copy numbers of CRC RT sequences in these chromosomes. Chromosomes with few or undetectable FISH signals were also observed in *C. canephora* (one pair), *C. eugenioides* (one pair), and *C. arabica* (two chromosome pairs).

**Table 3:** Cytogenetic distribution of CRC RT domains in *Coffea canephora, C. eugenioides*, and *C. arabica*.

**Chromosome Location**	**Chromosomal pairs with FISH signals**		
	***C. canephora***	***C. eugenioides***	***C. arabica***
Centromeric	7	7	9
Proximal & dispersed	1	2	7
Proximal & interstitial dots	1	0	3
Interstitial & dispersed	1	1	1
No signals	1	1	2
Total	11	11	22

**Figure 5:**
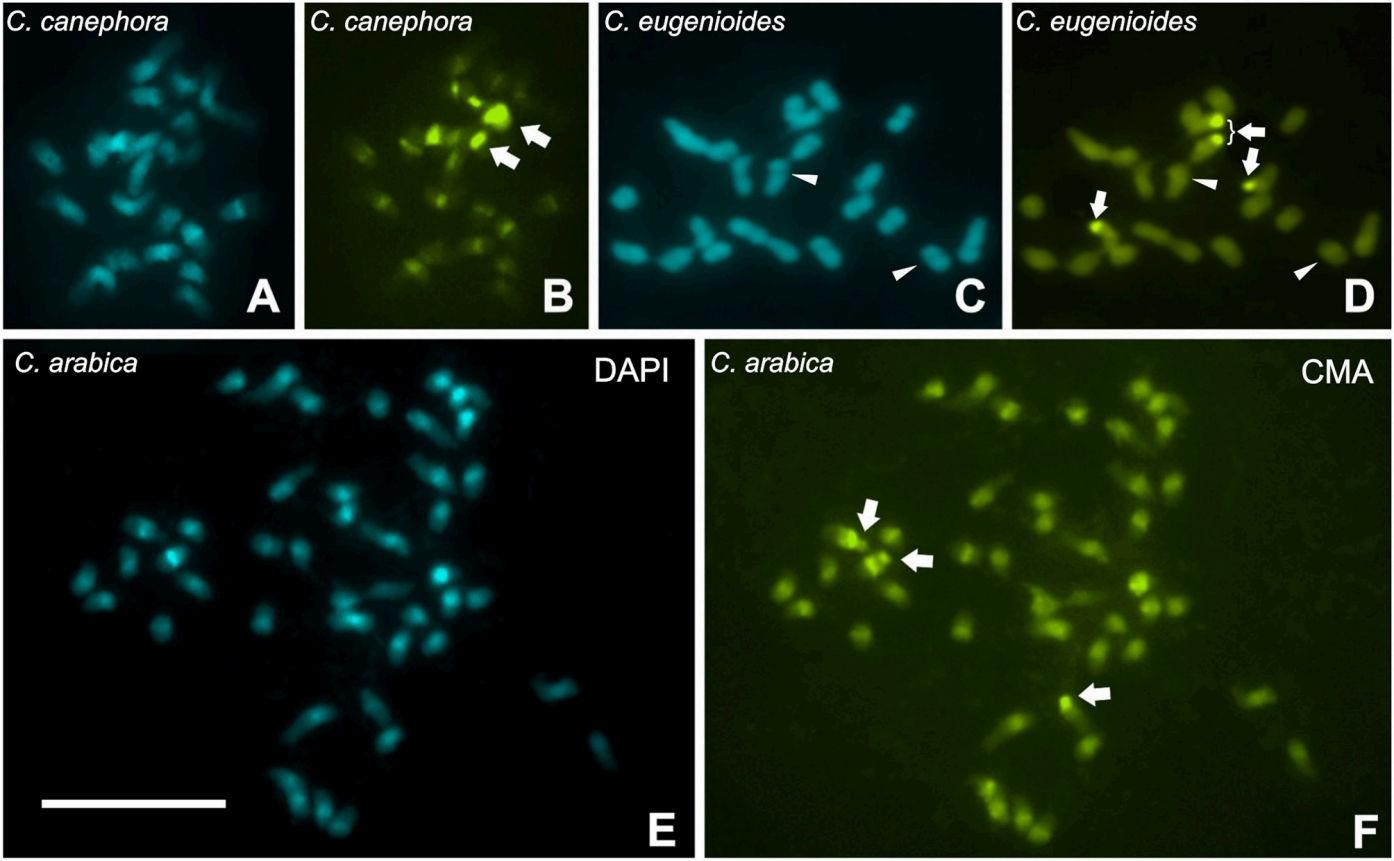
C-CMA/DAPI banding in *Coffea canephora* **(A,B)**, *C. eugenioides* **(C,D)**, and *C. arabica* **(E,F)**, showing an accumulation of C-CMA^+^/DAPI^+^ bands in the proximal regions of *C. canephora* and *C. arabica*, and absence of these bands in *C. eugenioides*. However, *C. eugenioides* seems to be inconspicuous C-CMA^+^/DAPI^−^ bands (thin bands of difficult visualization indicated as arrowhead), in the proximal regions of some chromosomes that are not present in the other two species **(C,D)**. Arrows indicate C-CMA^+^/DAPI^−^ bands accumulated in the terminal regions that are associated to nucleolar organizing regions (data not shown). Bar = 10 μm.

The C-CMA/DAPI banding indicated that C-CMA^+^/DAPI^−^ were associated to NOR bearing chromosomes in these three species. In *C. canephora* and *C. arabica*, C-CMA^+^/DAPI^+^ bands were accumulated in proximal regions (Figures 5A,B,E,F), while these bands were absent or few accumulated in *C. eugenioides* (Figures 5C,D). In this last species, C-CMA^+^/C-DAPI^−^ bands seem to be inconspicuous in the proximal regions of some chromosomes and absent in most of them (Figures 5C,D). These results showed also that C-CMA^+^ and C-DAPI^+^ heterochromatin can be co-localized with RT CRC hybridization signals for *C. canephora* and *C. arabica* chromosomes, but not for *C. eugenioides*.

### The *C. canephora* and *C. arabica* chromosome 5 putative centromeric regions are enriched of CRC elements

**Figure 6:**
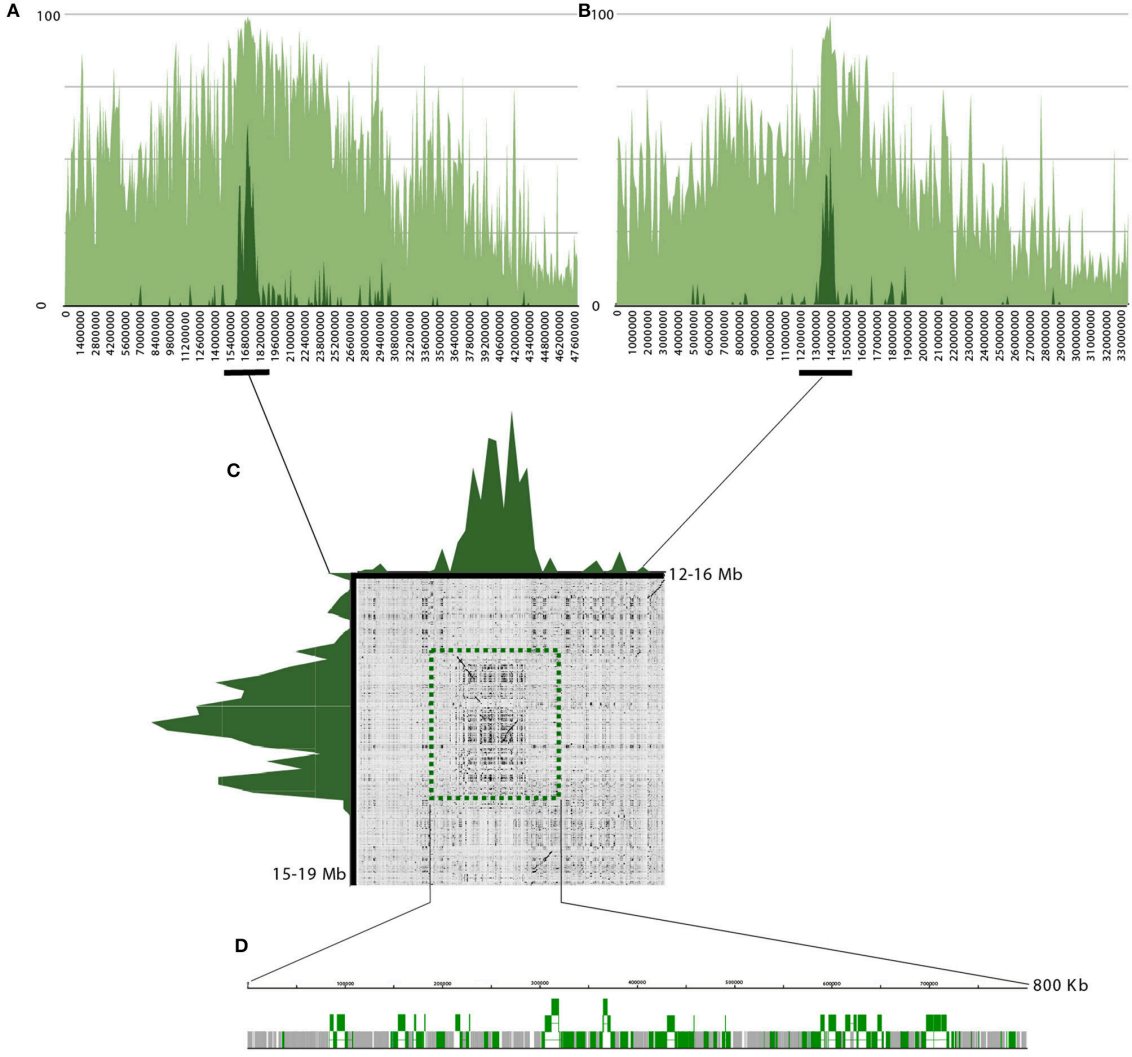
Structure and annotation of pseudo-chromosomes 5 from *C. canephora* and *C. arabica*. **(A)** Density of transposable elements (light green) and CR elements (dark green) of pseudo-chromosomes 5 from *C. canephora*. X-axis represents the density of elements en percentage and Y-axis the coordinates of the pseudochromosomes. **(B)** Density of transposable elements (light green) and CR elements (dark green) of pseudo-chromosomes 5 from the *C. canephora* sub-genome in *C. arabica* **(C)**. Dot-plot graphical view of sequence comparison of 4 Mb *in C. arabica* (horizontal) and *C. canephora* (vertical). Dark green peaks represent the density of CR elements in these regions. **(D)** Sequence organization of the 800 kb centromeric region in *C. arabica*. Gray blocks represent transposable elements and green blocks are CRC elements.

Based on the FISH data and localization of RT CRC on *C. canephora* genome sequences, the pseudochromosome 5 has been selected for further analysis. The density of transposable elements (light green, annotated on *C. canephora;* Denoeud et al., 2014) and full-length CRC elements (dark green) were displayed along the pseudochromosome 5 from *C. canephora* (Figure 6A) and along the pseudochromosome 5 sub-genome *C. canephora* from *C. arabica* (Figure 6B). Data showed a high density of CRC elements in the median part for both orthologous pseudochromosomes. A dot-plot of 4 Mb length around these regions in *C. canephora* and *C. arabica* (Figure 6C), suggest a conservation where CRC elements density (dark green) is the highest. Annotations of highest density regions containing CRC elements of *C. canephora* and *C. arabica*, with 1.2 Mb and 800 kb length, respectively (Figure 6D), revealed that 94.1 and 91.7% of these regions consisted of transposable elements. LTR retrotransposons and non-autonomous derivatives represent 84.4 and 79.7% and CRC elements represent 33.7 and 35% in *C. canephora* and *C. arabica*, respectively, whereas transposons account for 0 and 0.7%. Interestingly, the CRC family H, represents alone 17.84 and 25.28 of the analyzed regions in *C. canephora* and *C. arabica*, suggesting a local enrichment. Beside CRC, the Del lineage is the most redundant with 15.9 and 9.6%. A detailed annotation was performed for the centromeric region of *C. arabica* pseudochromosome 5. Ninety-one complete or partial CRC elements were annotated for which 76 fell into the H family. Twenty-three complete and 13 putative non-autonomous CR elements carrying both intact LTR ends were recovered and their insertion times were estimated. Seventeen of them have a very recent insertion time (>1 Mya), similarly to estimation at the genome scale (Supplemental data 7). In these regions rich in CRC elements, no tandem repeats were observed in *C. canephora* and in *C. arabica* assembled sequences. Insertion of CRC elements into tandem arrays were directly searched in raw *C. canephora* PacBio reads, before their assembly, using BLAST and dot-plot. Here again no tandem repeats associated with CRC elements of the H family were found. The density of transposable elements (light green, annotated on *C. canephora;* Denoeud et al., 2014) and full-length CRC elements (dark green) were also displayed along all pseudochromosome from *C. canephora, C. arabica and C. eugenioides* (Supplemental datas 9–11). Most of the pseudochromosomes showed a clear peak of accumulation.

## Discussion

### Characterization of CRC elements in *Coffea* yields 10 distinct groups

Despite numerous centromeric retrotransposons elements identified in monocot and dicot species (Neumann et al., 2011), their diversity and classification into types, as well as their respective contribution to the structure of centromeric regions is poorly known for most higher plant groups. In this study, we identified 10 groups of Centromeric Retrotransposons of *Coffea* (CRC) in the genomes of *C. arabica*, an allotetraploid species and its two diploid parents, *C. canephora* and *C. eugenioides*. This work was based on high coverage of PacBio reads used for *C. arabica, C. canephora*, and *C. eugenioides* genomes produced by the ACGC (Mueller et al., 2015). Centromeric retrotransposons in plants were initially organized into three groups, based on the presence of a CR domain extending into the 3′ LTR and a chromodomain at the C terminus of the POL polyprotein (Neumann et al., 2011). In *Coffea* the 10 identified groups fall into two of these groups: those possessing a CR motif (most of them, group “A” from Neumann et al. (2011), corresponding to our B, C, D, E, F, G, H, X, and Y groups) and those carrying a terminal chromodomain-like (group “C” from Neumann et al. (2011), corresponding to our A group). These data indicate that centromeric retrotransposons could be more diverse in plants than previously proposed by Neumann et al. (2011).

Chromodomain might target integration of chromovirus LTR retrotransposons into heterochromatic chromosome regions (Novikova, 2009), and these specificities could allow the *CRM* accumulation into proximal chromosome regions, such as in *Coffea*, or may be still associated with epigenetic mechanisms (Houben et al., 2007; Neumann et al., 2011). However, most of CRC groups (B, Y, C, E, D, F, G, X, and H) that are similar to the “C” group of Neumann et al. (2011), did not have any chromodomain nor zinc finger domains, but carried a CR motif. This motif appears particularly important for centromeric retrotransposons to target the heterochromatin (Gao et al., 2008), but they are probably not associated with epigenetic changes in H3 histones (Neumann et al., 2011). The “B” group of centromeric retrotransposons, as defined by Neumann et al. (2011), without CR motif nor chromodomain, was not identified in the autonomous elements set in *C. arabica, C. canephora*, and *C. eugenioides* genomes. This group has been probably lost or degenerated during the evolution of the *Coffea* genus, since group “B” was identified in other dicotyledonous, such as *Vitis, Arabidopsis, Medicago*, and *Populus* (Neumann et al., 2011). Another possibility is that the group B of Neumann has been lost or degenerated earlier during the evolution of the Rubiaceae family or the Asterids branch of dicots, because the genera previously mentioned belong to the Rosids branch.

Non-autonomous centromeric retrotransposons identified in *Coffea* belong to different families: (TRIM, Witte et al., 2001), (LARD, Kalendar et al., 2004), or lacking the POL polyprotein region such as TR-GAG (Chaparro et al., 2015). This last family was also found in rice (Nagaki et al., 2005). Non-autonomous CRC shared similarities with the nine autonomous CRC groups containing CR motif, suggesting a direct relationship between autonomous and non-autonomous elements, as well as they could indicate that non-autonomous CRC may use the enzymatic machinery of complete elements for their own mobility (Wicker et al., 2007).

### *In silico* copy numbers and insertion time of CRC families

The *C. arabica* genome contains a higher number of complete CRC copies than the related diploid *C. canephora* or *C. eugenioides* genomes, and it is in accordance to relationships between the polyploidization and copy number variation observed for other retrotransposons in allopolyploid genomes (Parisod et al., 2010). However, the cumulative number of CRC copies is higher for the two diploid than for the allotetraploid species, suggesting that changes occurred either during the hybridization steps leading to *C. arabica* or very recently, after the hybridization. CRC groups may have been amplified very recently in these three genomes, but with higher amplitude in *C. canephora* during the last million years. However, it remains unclear if the CRC copy number variation is only due to differential rates of amplification or if this variation is due to an efficient process of elimination via unequal or illegitimate recombination (Bennetzen, 2007). Two groups with the highest copy number (B and H) in the three species also showed recent peaks of insertion time, suggesting they were amplified recently in the *Coffea* genomes. The only exception is the B group of *Coffea*, which seems to have an ancient origin in *C. arabica*. The number of *C. arabica* CRC observed in present days compared to its progenitors should be carefully interpreted, because the present germplasms of *C. canephora* and *C. eugenioides* studied recently can have accumulated some differences in relation to those which gave rise to the amphidiploidy in *C. arabica*. In addition, we have also to consider that the worldwide *C. arabica* collection had been originated from a few Ethiopian individuals (Carvalho, 1946), and they have been extensively submitted to agronomic breeding selection.

### The E and H CRC groups target putative centromeric regions in *Coffea*

Along plant chromosomes, *Copia* and *Gypsy* superfamilies can be found distributed in blocks and scattered (Lopes et al., 2013; Santos et al., 2015; Zhang et al., 2017). One notable exception is the *Gypsy* Centromeric Retrotransposon lineage, located preferentially into centromeric and proximal regions (Du et al., 2010; Sharma and Presting, 2014). In *Coffea* species, the distribution of CRC families showed two contrasting situations. One family, the B group, appears scattered along *C. canephora* pseudochromosomes, whereas the H and, in a lesser extent, the E group, appeared clustered into proximal chromosome regions, as expected for Centromeric Retrotransposons (Sanseverino et al., 2015).

Although it was possible to separate 10 CRC groups using complete sequences, the high identity (>90%) of RT regions made difficult the design of specific primers for each group. While specific FISH for each CRC family was impossible with RT-domains, other and more divergent regions such as LTR or GAG gave inaccurate results.

Results of FISH using a generic RT-CRM probe is in agreement with a targeting of chromodomain and CR motif into centromeric regions associated to CENH3 (Houben et al., 2007; Neumann et al., 2011; Li et al., 2013), suggesting an interaction between these elements and centromeric proteins.

Our cytological observations suggested that the hybridization profile is variable among species and chromosomes in *Coffea*. In *C. eugenioides*, FISH signals were strictly associated to centromeric regions, whereas in *C. canephora* and *C. arabica* signals appear less specific to centromeres, and scattered along interstitial regions. This could be the result of a small CRC RT copy numbers hybridized. We hypothesize the two pairs without bright signals in *C. arabica* could be homologous chromosomes to those without FISH signals from the parental genomes (one pair each). Scattered FISH signals using CR probe were also reported in *Saccharum spontaneum* (Zhang et al., 2017). Surprisingly one chromosome pair in *C. canephora* and *C. eugenioides* and two in *C. arabica* did not exhibit evident centromeric signals. All these variable hybridization patterns could be associated also with differential occurrence of proximal C-CMA^+^/DAPI^+^ bands, that were observed in *C. canephora* and *C. arabica*, and absent or difficult to distinguish in *C. eugenioides*. The heterochromatin accumulation may be associated with increase and expansion of CRC elements beyond the centromere toward the interstitial regions observed in *C. canephora* and *C. arabica*. However, additional tests are necessary to confirm this assumption, especially in relation to equilocal dispersion (Schweizer and Loidl, 1987) of repetitive DNA families into proximal regions of *Coffea* chromosomes. In addition, it is possible that, CR elements containing the 3′ terminal CR motif, and that represent a fraction of the all CR families, would be more likely inserted into the putative centromeric regions, while the other CRCs (lacking the CR motif) could be less specific and occupy other chromosomal regions.

CRC elements carrying a CR motif may also present diverse pattern of insertion, i.e., they can be specific to putative centromeric regions (E and H groups) and/or to interstitial regions (B group). The presence of the CR motif may be not the *sine qua non* condition for a putative centromeric targeting and that other mechanisms may intervene for chromosomal regions targeting by chromoviruses in plants. Chromatin immunoprecipitation followed by sequencing (ChIP-Seq) using antibodies against the centromere-specific histone H3 of Coffee are now required to validate putative centromeric regions as active centromeres.

### The putative centromeric region of chromosome 5 is mainly composed of the H family

Repetitive DNA families, such as centromeric retrotransposons and tandem repeats, participate in the complex organization of centromeric regions, especially of the kinetochore formation (Neumann et al., 2011). In *Coffea*, 23 CRCs were predicted as elements that have some role in the centromeric regions, as observed in other plant groups (Han et al., 2010; Sanei et al., 2011). However, it has not been yet clarified what CRC types (complete, truncated, partial, or non-autonomous) may participate in kinetochore formation. The presence of partial and truncated elements on proximal chromosome regions suggests that unequal and illegitimate recombination mechanisms may also act on centromeric regions in a neutral manner (Bennetzen, 2007). CR elements were frequently associated with satellite DNA repeats in centromeric regions of other plant species (Cheng et al., 2002; Lim et al., 2007), except for the wheat chromosome 3B, only composed of CRW retrotransposons families (Li et al., 2013). This observation may suggest that CR elements alone might be sufficient to ensure the kinetochore function. But more detailed annotations and validation of centromeric regions of Coffee trees are necessary to understand the composition and the evolution of such critical chromosomal regions.

The diversity in types and chromosomal insertions of CRCs gave a more complex view of the structure and evolution of centromeric regions in *Coffea*, especially in relation to LTR-RTs along hybridization process. *C. arabica* showed an accumulation of proximal heterochromatin associated with more dispersed CRC profile on the chromosomes, suggesting that the roles and effects of centromeric retrotransposons can extend beyond the proximal domains. In the near future, the characterization of centromere sequences in diploid and allotetraploid *Coffea* genomes will bring more insights into the evolution of these chromosomal regions that play a crucial role in the cell life cycle.

## Author contributions

AV and RG: directed researches; RdCN and PY: performed FISH and bioinformatics and SO-A performed bioinformatics; PD, CF, and DM: performed sequencing and LM and SS performed genome assembly; AV, RG, AdK, and DC: wrote the manuscript.

### Conflict of interest statement

The authors declare that the research was conducted in the absence of any commercial or financial relationships that could be construed as a potential conflict of interest.
