# Supplementary material for: Structure and Distribution of Centromeric Retrotransposons at Diploid and Allotetraploid Coffea Centromeric and Pericentromeric Regions
Source: Front Plant Sci. 2018 Feb 15;9:175. doi: 10.3389/fpls.2018.00175 (PMC5818461; doi:10.3389/fpls.2018.00175)
Supplement: Supplementary file 1 [file DataSheet1.DOCX]

| **Organism** | **sequencing chemistry** | **collection protocol [min]** | **SMRT cell nb** | **output [Gb]** | **Coverage** | **PF read nb** | **PF Polymerase Read N50 [bp]** |
| --- | --- | --- | --- | --- | --- | --- | --- |
| *Coffea arabica* | P4/C2 and P5/C3 | 180 | 174 | 72 | 56x | 7,246,499 | 10,610 |
| *Coffea canephora* | P6/C4 | 240 | 65 | 55 | 77x | 3,721,573 | 16,988 |
| *Coffea eugenoides* | P6/C4 | 240 | 60 | 39 | 58x | 3,219,296 | 15,481 |

**Supplemental data 1**. Description of genome sequencing specification.

**Supplemental data 2.** Number of LTR-retrotransposons elements in the different *Gypsy* lineages in *Coffea arabica*, *C. canephora* and *C. eugenioides* as detected by LTR_STRUC.


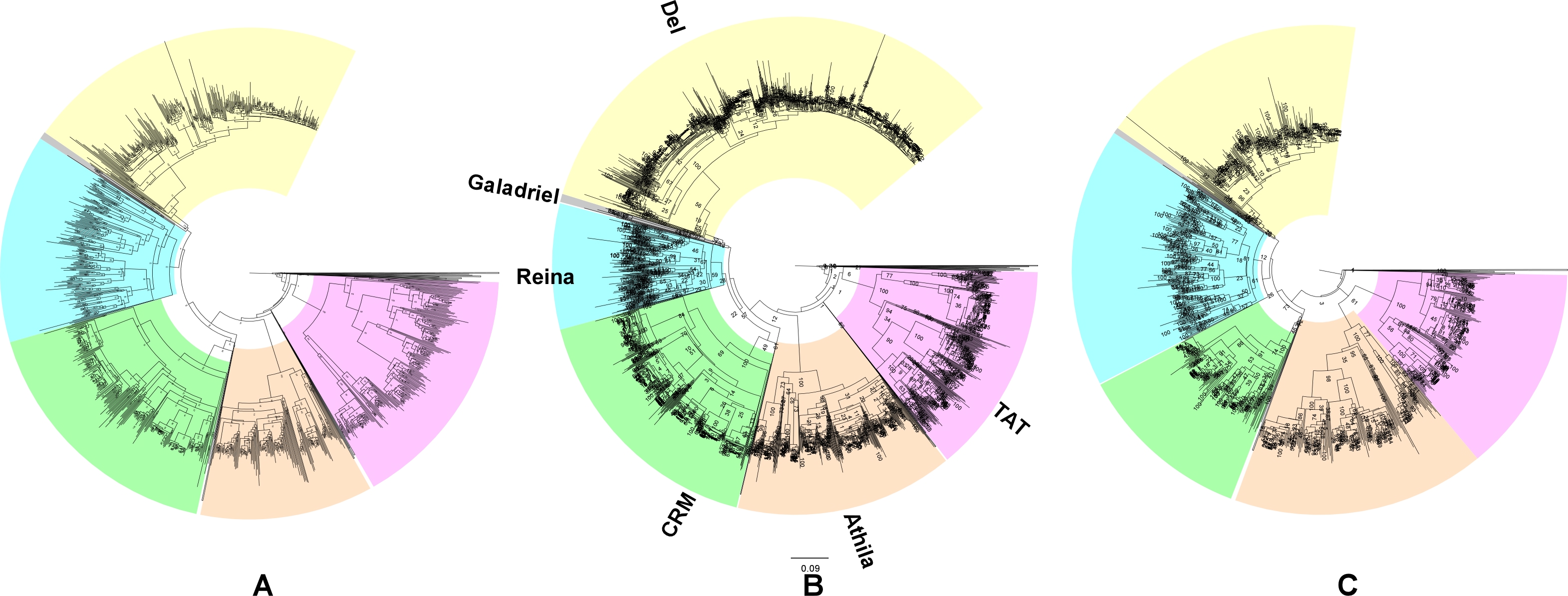


**Supplemental data 3.** RT-based phylogenetic analysis of *Gypsy* LTR-retrotransposons predicted in *Coffea arabica* (A), *C. canephora* (B) and *C. eugenioides* (C) identified by LTR_STRUC. The names of *Gypsy* lineages are indicated. Phylogenetic trees were based on protein alignments of Reverse Transcriptase domains. 1,226, 2,222 and 950 recovered domains were used for *C. arabica*, *C. canephora* and *C. eugenioides.*


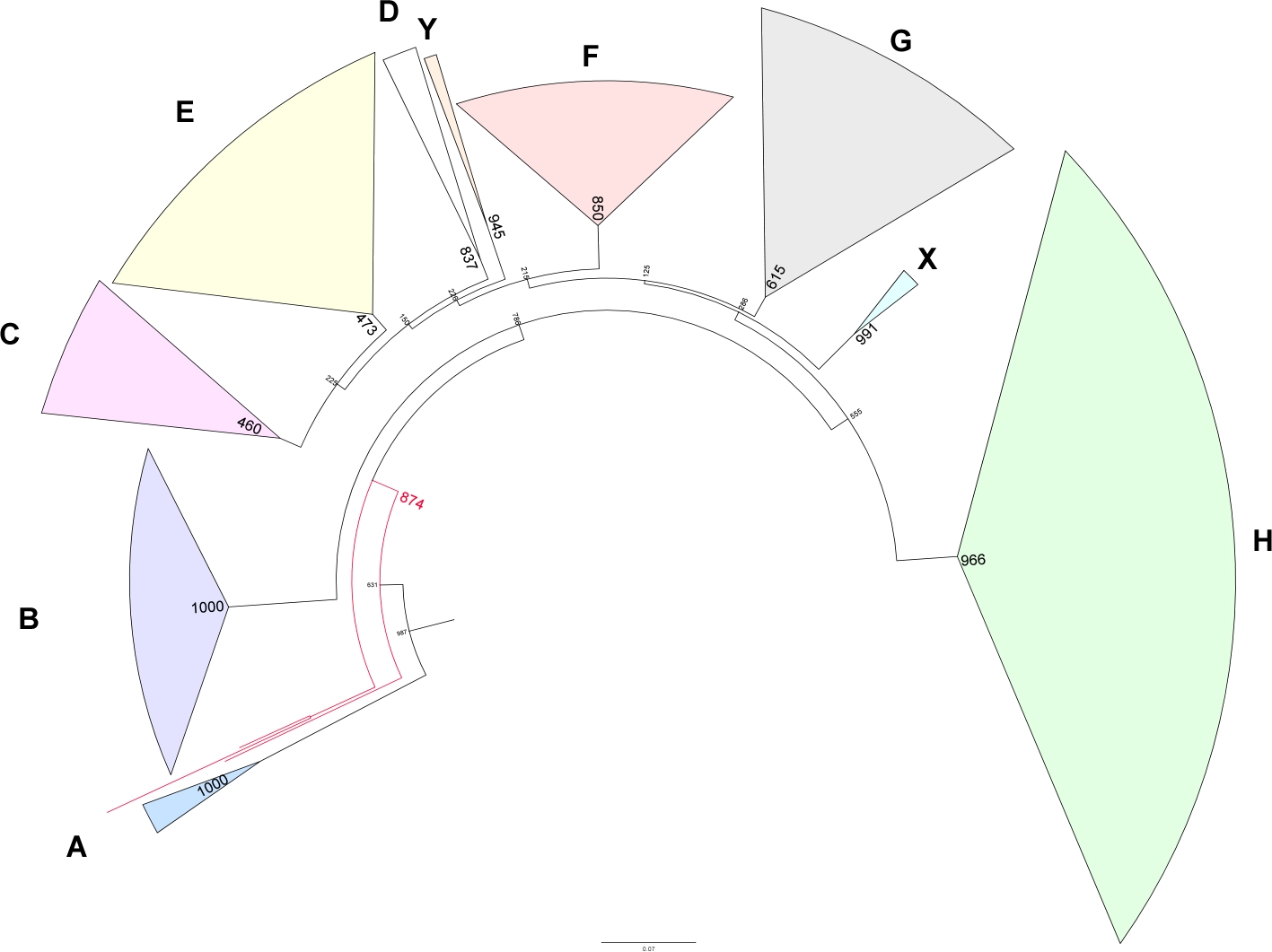


**Supplemental data 4.** NJ Phylogenetic tree of RT domains from 604 autonomous CRC elements from *Coffea canephora*, *C. eugenioides* and *C. arabica*.

Colors represent the 10 CRC groups as follow: A, B, C, D, E, F, G, H, Y and X. In red are represented the branch of representative *CRM* RT domains from the Gypsy DB (*CRM*, Beetle1 and Cereba). Bootstraps were indicated.

**Supplemental data 5.** Distribution of CRC groups on the *Coffea eugenioides*, *C. canephora* and *C. arabica* predicted complete elements by LTR_STRUC.

| **Genomes** | **Absolute numbers and CRC groups** | | | | | | | | | |
| --- | --- | --- | --- | --- | --- | --- | --- | --- | --- | --- |
|  | **A** | **B** | **Y** | **C** | **E** | **D** | **F** | **G** | **X** | **H** |
| **CRcc** | 5 | 68 | 0 | 16 | 53 | 6 | 43 | 53 | 0 | 91 |
| **CRce** | 2 | 20 | 0 | 11 | 10 | 0 | 7 | 0 | 2 | 31 |
| **CRca** | 0 | 1 | 2 | 2 | 15 | 0 | 21 | 8 | 3 | 49 |
| **Genomes** | **Relative numbers (%) and CRC groups** | | | | | | | | | |
|  | **A** | **B** | **Y** | **C** | **E** | **D** | **F** | **G** | **X** | **H** |
| **CRcc** | 1.49 | 20.30 | 0 | 4.78 | 15.82 | 1.79 | 12.84 | 15.82 | 0 | 27.16 |
| **CRce** | 2.41 | 24.10 | 0 | 13.25 | 12.05 | 0 | 8.43 | 0 | 2.41 | 37.35 |
| **CRca** | 0 | 0.99 | 1.98 | 1.98 | 14.85 | 0 | 20.79 | 7.92 | 2.97 | 48.51 |

The following letters: A. B. C. D. E. F. G. H. X and Y correspond to CRC groups, as for instance: CRcc_group_A. CRcc = centromeric retrotransposons of *C. canephora*; CRce = centromeric retrotransposons of *C. eugenioides*; CRca = centromeric retrotransposons of *C. arabica*.


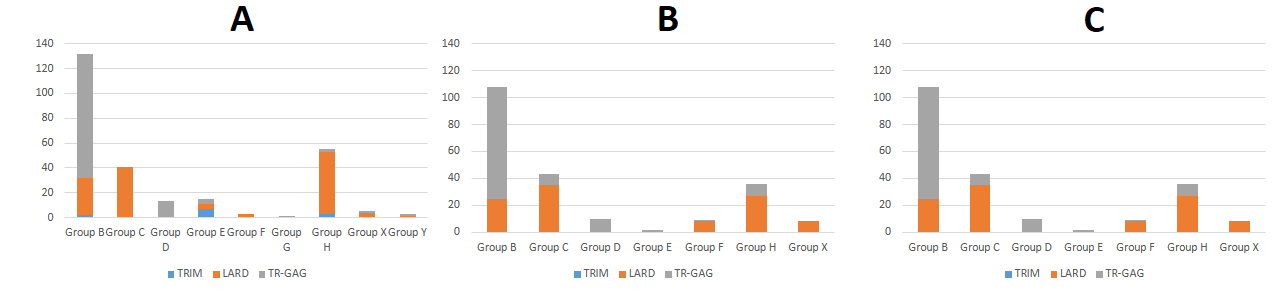


**Supplemental data 6.** Copy number of putative non-autonomous CRC elements (TRIM, LARD and TR-GAG) in *Coffea arabica, C. canephora and C. eugenioides*.

Up: Copy number of non-autonomous CRC elements for each group.

Down: Copy number of each type of non-autonomous elements for each CRC groups A) *C. canephora* B) *C. eugenoides* and C) *C. arabica.*

**Supplemental data 7**. Estimation of insertion times of CRC groups in *Coffea arabica, C. canephora and C. eugenioides*.

A. Insertion times of all CRC groups in all genomes. B. Insertion times of each group in *C. canephora*. C. Insertion times of each group in *C. arabica.* D. Insertion times of each group in *C. eugenioides.* Insertion times were estimated using a substitution rate of 1.3x10^-8^ (Ma and Bennetzen 2004). All X-axis represent Insertion times in million years and all Y-axis represent the number of full-length elements.

Forward ACTGTCGGGCTGTAAATGCT.........|.........|.........|.........|.........|.........|.........|.........100

Primer position

CRcc_group_A ATAGCCATGCAATCAACAAGATAACAATCAAGTACAGGTTTCCCATACCCAGACTTGATGATATGATTGATATGATGGCTGGGTCAACTATCTACACTAA

CRce_group_A ATAGCCGTGCAATCAATAAGATAACAATCAAGTATAGGTTTCCCATACCTAGATTTGATAATATGATTGATATGATGACTGGTACTATTGTCTACACTAA

CRcc_group_B ATTGTCGTGCAATTAATAAAATCACTGTTAAGTATCGTCATCCCATTCCTAGGTTAGACGATATGTTAGAAGAATTGCATGGGGCAATTATTTTCACTAA

CRce_group_B ATTGTCGTGCAATTAATAAAATTACTGTCAAGTATCGTCATCCTATTCCTAGGTTAGATGATATGTTAGAAGAATTGCATGGGGCAATCATTTTCACTAA

CRca_group_B ATTGTCGTGCAATTAATAAAATTACTGTCAAGTATCGTCATCCTATTCCTAGGTTAGATGATATGTTAGAAGAATTGCATGGGGCAATCATTTTCACTAA

CRca_group_Y ACTGCCGAGCTACCAATACCATAACGGTAAAGTATCGTCATCTCATCCCTCATCTAGATGACATGTTAGATGAATTACACGGGGCAATTATATTCACAAA

CRcc_group_C ATTGTAGAGCCGTAAATGCCATCACGGTAAAGTATCGCCACCCTATACCTCGCTTAGATGACATGCTTGATGAGTTACATGGGGCTGTGTTCTTTACCAA

CRce_group_C ATTGTAGGGCCGTAAATGCCATCACGGTAAAGTATCGTCACCCTATACCTCGCTTAGATGACATGCTTGATGAGTTACATGGGGCTGTGTTCTTTACCAA

CRca_group_C ATTGCAGAGCCGTAAATGCCATCACGGTAAAGTATCGCCACCCTATACCTCGCTTAGATGACATGCTTGATGAGTTACATGGGGCTGTGTTCTTTACCAA

CRcc_group_E ACTGTAGGGCTGTTAATGCAATAACGGTAAAATATCGTCACCCTATTCCTAGACTTGATGATATGTTAGATGAGCTATATGGTGCTGTGATTTTCACTAA

CRce_group_E ACTGTAGGGCCGTTAATGCAATAACGGTAAAATATCGTCACCCTATTCCTAGACTTGATGATATGTTAGATGAGCTATATGGTGCTGTGATTTTCACTAA

CRca_group_E ACTGTAGGGCTGTTAATGCAATAACGGTAAAATATCGTCACCCTATTCCTAGACTTGATGATATGTTAGATGAGCTATATGGTGCTGTGATTTTCACTAA

CRcc_group_D ACTGCCGAGCTGTCAACGCCATCACAGTTAAATATCGTCACCCTATATCTCGTTTAGATGATATACTTGACGAATTATATGGTGCAGTCATATTCACTAA

CRcc_group_F ACTGTCGAGCAGTCAATGCAATCACGGTAAAATATCGTCACCCTATCCCTAGGTTAGATGATATGTTAGATGAATTGCATGGTGCCATTATATTTACTAA

CRce_group_F ACTGTCGAGCAGTCAATGCAATCACGGTAAAATATCGTCACCCTATCCCTAGGTTAGATGATATGTTAGATGAATTGCATGGTGCCATTATATTCACTAA

CRca_group_F ACTGTCGAGCAGTCAATGCAATCACGGTAAAATATCGTCACCCTATCCCTAGGTTAGATGATATGTTAGATGAATTGCATGGTGCCATTATATTTACTAA

CRcc_group_G ATTGTAGAGCCATAAATGCCATAACGGTAAAGTATCGCCATCCCATACCTCGATTAGATGACATGCTTGATGAGTTACATGGTGCTATTATATTTACTAA

CRca_group_G ATTGTAGAGCCATAAATGCCATAACGGTAAAGTATCGCCATCCCATACCTCGATTAGATGACATGCTTGATGAGTTACATGGTGCTATTATATTTACTAA

CRce_group_X ATTGTCGTGCCATTAACGCCATCACGATAAAGTATCGCCATCCTATACCACGTTTAGATGACATGCTTGATGAACTTCATGGTGCCGTTATTTTCACTAA

CRca_group_X ATTGTCGTGCCACTAATGCCATCACGGTAAAGTATCACCATCCTATACCACGTTTAGATGACATGCTTGATGAACTTCATGGTGCCGTTATTTTCACTAA

CRcc_group_H ACTGTCGGGCTGTGAATGCCATCACTGTCAAATATCGGCATCCCATCCCTCGACTTGATGATATGCTGGACGAACTCGACGGTGCCATTATTTTCACCAA

CRce_group_H ACTGTCGGGCTGTGAATGCTATCACTGTTAAATATCGTCATCCCATTCCTAGACTTGATGATATGCTTGATGAACTCGATGGGGCCATTATTTTCACCAA

CRca_group_H ACTGTCGGGCTGTGAATGCTATCACTGTCAAATATCGGCATCCCATCCCTCGACTTGATGATATGCTGGACGAACTCGACGGTGCCATTATTTTCACCAA

CRcc_group_A AATTGACTTGCGAAAGGGGTACTATCAAATCAGAATTCGTCCAGGTGATGAGTGGAAAACAGCCTTTGAGACGAAGGATGGTCTCTATGAGTGGCTAGTG

CRce_group_A AATTGACTTGCGAAAGGGGTACTGTTAGATCAGAATTCGTTCAGGTGATGAGTGGAAAACAGCCTTCAAGGCGAAGGATGGTCTCTACGAGTGGCTAGTG

CRcc_group_B AATTGATTTAAGATCTGGGTATCATCAATTAAGGATAAAGGAAGGTGACGAATGGAAAACTGCTTTCAAAACAAAGTATGGTCTTTATGAGTGGCTTGTG

CRce_group_B AATTGACTTAAGATCTGGTTATCATCAAATAAGGATAAAGGAAGGAGACGAATGGAAAACTGCTTTCAAAACAAAGTATGGTCTTTATGAGTGGCTTGTG

CRca_group_B AATTGACTTAAGATCTGGTTATCATCAAATAAGGATAAAGGAAGGAGACGAATGGAAAACTGCTTTCAAAACAAAGTATGGTCTTTATGAGTGGCTTGTG

CRca_group_Y AATTGATCTGAAATCTGGATATCATCAAATTAGAATTAAAGAGGGGGACGAATGGAAGACTGCATTTAAGACTAAGTACGGGTTATATGAGTAGTTAGTC

CRcc_group_C AATTGATCTCAAAAGTGGATACCATCAAATTAGGATTAAGGAGGGGGATGAATGGAAAACTGCCTTCAAAACTAAGTATGGATTGTATGAGTGGTTAGTG

CRce_group_C AATTGATCTCAAAAGTGGGTACCATCAAATTAGGATTAAGGAGGGGGATGAATGGAAAACTGCCTTTAAAACTAAGTATGGATTGTATGAGTGGTTAGTG

CRca_group_C AATTGATCTCAAAAGTGGGTACCATCAAATTAGGATTCAGGAGGGGGATGAATGGAAAACTGCCTTCAAAACTAAGTATGGCTTGTATGAGTGGTTAGTG

CRcc_group_E AATTGATCTAAAAAGCGGTTATCATCAAATTAGGATGAAAGAGGGGGATGAATGGAAAACGGCCTTTAAAACCAAACATGGCTTGTACGAGTGGTTAGTT

CRce_group_E AATTGATCTAAAAAGCGGTTATCATCAAATTAGGATGAAAGAGGGGGATGAATGGAAAACGGCCTTTAAAACCAAACATGGCTTGTACGAGTGGTTAGTT

CRca_group_E AATTGATCTAAAAAGCGGTTATCATCAAATTAGGATGAAAGAGGGGGATGAATGGAAAACGGCCTTTAAAACCAAACATGGCTTGTACGAGTGGTTAGTT

CRcc_group_D AATAGATCTTAAGAGTGGCTACCATCAAATTCGAATGAAAGAAGGTGATGAGTAGAAGACAACCTTCAAGACTAAGTATGGTCTGTATGAGTGGCTAGTT

CRcc_group_F AATTGATTTGAAAAGTGGTTACCACCAAATAAGAATGAAAGAGGGAGATGAGTGGAAAACCGCTTTCAAAACTAAACATGGTCTATACGAGTGGTTAGTA

CRce_group_F AATTGATTTGAAAAGTGGTTACCACCAAATAAGAATGAAAGAGGGAGATGAGTGGAAAACCGCTTTCAAAACTAAACATGGTCTATATGAGTGGTTAGTA

CRca_group_F AATTGATTTGAAAAGTGGTTACCACCAAATAAGAATGAAAGAGGGAGATGAGTGGAAAACCGCTTTCAAAACTAAACATGGTCTATACGAGTGGTTAGTA

CRcc_group_G GATTGACTTGAAAAGTGGTTATCACCAAATACGCATGAAAGAAGGGGATGAGTGGAAAACAGCATTTAAAACAAAACATGGTCTGTATGAGTGGTTGGTC

CRca_group_G GATTGACTTGAAGAGTGGTTATCACCAAATACGCATGAAAGAAGGGGATGAGTGGAAAACAGCATTTAAAACAAAACATGGTCTGTATGAGTGGTTGGTC

CRce_group_X AATTGATCTTAAAAGTGGTTATCATCAAATTAGGATGAAAGAAGGAGATGAATGGAAAACTGCATTTAAGACTAAACATGGTTTGTATGAGTGGTTAGTC

CRca_group_X AATTGATCTTAAAAGTGGTTATCATCAAATTAGGATAAAAGAAGGAGATGAATGGAAAACTGTATTTAAGACTAAACATGATTTGTATGAGTGGTTAGTC

CRcc_group_H AATTGACTTGAGGAGTGGCTATCATCAAATCCGAATGAAGGAAGGCGATGAGTGGAAGACGGCCTTCAAAACCAAACATGGTCTCTATGAGTGGCTGGTC

CRce_group_H AATTGACTTAAGGAGTGGCTATCATCAAATTAGGATGAAAGAAGGTGATGAGTGGAAAACAGCCTTCAAAACCAAACATGGTCTCTATGAGTGGCTAGTC

CRca_group_H AATTGACTTGAGGAGTGGCTATCATCAAATCCGAATGAAGGAAGGCGATGAGTGGAAGACGGCCTTCAAAACCAAACATGGTCTCTATGAGTGGCTAGTC

.........|.........|.........|.........|.........|.........|.........|.........|.........|.........200

CRcc_group_A ATGCCATTTGGCCTCTCTAATGCTCCCAGCACCTTTATGAGATTCATGACGCGGGTATTTCAGCCCTTTATTGGCCATTTTTTGGTTGTCTATTTCGATG

CRce_group_A ATGCCGTTTGGCCTCTCTAATGCTCCTAGCACCTTTATGAGATTCATGACACAGGTGTTTCAGCCCTTTATTGGCCTTTTTTTGGTTGTCTATTTCGATG

CRcc_group_B ATGCCTTTTGGCTTAACAAACGCTCCAAGTACTTTCATGAGATTAATGAACCATGTTTTAAGGCATTTTCTTGGGAAGTTCGTTGTTGTTTATTTTGATG

CRce_group_B ATGCCTTTCGGCTTAACAAACGCTCCAAGTACTTTCATGAGGTTAATGAACCATGTTTTAAGGCATTTTCTTGGGAAGTTTGTTGTTGTTTATTTTGATG

CRca_group_B ATGCCTTTCGGCTTAACAAACGCTCCAAGTACTTTCATGAGGTTAATGAACCATGTTTTAAGGCATTTTCTTGGGAAGTTTGTTGTTGTTTATTTTGATG

CRca_group_Y ATACCTTTTGGCCTTACTAATGCACCAAGCACCTTTATGCGACTAATGAACCATGTCTTGCGTGAGTACTTAGGCAAATTTGTAGTAGTATATTTTGATG

CRcc_group_C ATGCCTTTTGGGCTGACTAATGCACCTAGTACCTTCATGAGATTAATGAACCATGTTCTACGTCCATTCTTAGGGAAATTTGTGGTAGTTTATTTTGACG

CRce_group_C ATGCCTTTTGGGCTCACTAATGCACCTAGCACCTTCATGAGGTTAATGAACCATGTTCTGCGTCCATTCTTAGGAAAATTCGTGGTAGTTTACTTTGATG

CRca_group_C ATGCCTTTTGGGCTAACTAATGCACCTAGTACCTTCATGAGGTTAATGAATCATGTTCTGCGTCCATTCTTAGGAAAATTTGTGGTAGTTTACTTTGACG

CRcc_group_E ATGCCATTTGGACTAACTAATGCACCTAGTACGTTCATGAGGTTGATGAACCATGTACTTCGTCCATTCCTTGGAAAATTTGTAGTTGTATATTTTGACG

CRce_group_E ATGCCATTTGGACTAACTAATGCACCTAGTACGTTCATGAGATTGATGAACCATGTACTTCGTCCATTCCTTGGAAAATTTGTAGTTGTATATTTTGACG

CRca_group_E ATGCCATTTGGACTAACTAATGCACCTAGTACATTCATGAGGTTGATGAACCATGTACTTCGTCCATTCCTTGGAAAATTTGTAGTTGTATATTTTGACG

CRcc_group_D ATACCTTTTGGTTTGACTAATGTTCCTAGTACATTTATGCACTTTATGAACCATGTGCTTCGCCCTTTCTTAAGTAAATTTGTTGTTGTTTATTTTGACG

CRcc_group_F ATGCCATTTGGTTTAACTAATGCACCTAGTACCTTCATGCGTTTAATGAACCATGTTTTGAGACCTTTTCTTGGGAAATTTGTAGTGGTTTACTTTGATG

CRce_group_F ATGCCATTTGGCTTAACTAATGCACCTAGTACCTTCATGCGTTTGATGAACCATGTTTTGAGACCTTTTCTTGGGAAATTTGTAGTGGTTTACTTTGATG

CRca_group_F ATGCCATTTGGTTTAACTAATGCACCTAGTACCTTCATGCGTTTAATGAACCATGTTTTGAGACCTTTTCTTGGGAAATTTGTAGTGGTTTACTTTGATG

CRcc_group_G ATGCCTTTTGGCTTAACTAATGCACCTAGTACCTTCATGAGATTAATGAACCATGTTTTGCGTTCTTTTATTGGTAAATTTGTGGTAGTCTACTTTGATG

CRca_group_G ATGCCTTTTGGCTTAACTAATGCACCTAGTACCTTCATGAGATTAATGAACCATGTTTTGCGTTCTTTTATTGGTAAATTTGTGGTAGTCTACTTTGATG

CRce_group_X ATGCCATTTGGCCTGACTAATGCCCCTAGTACTTTCATGCGTTTAATGAACCATGTCTTACGTGCGTTCTTGGACAAATTTGTGGTAGTCTACTTTGATG

CRca_group_X ATGCCATTTGGCCTGACTAATACCCCTAGTACCTTCATGCGTTTGATGAACCATGTCTTACGTGCTTTCTTGGACAAATTTGTGGTAGTCTACTTCGATG

CRcc_group_H ATGCCTTTTGGCTTGACCAATGCCCCTAGCACCTTTATGCGCCTCATGAACCATGTCTTGAGACACTTCATTGGTAAATTCGTCATTGTTTACTTTGATG

CRce_group_H ATGCCTTTTGGCTTGACTAATGCTCCTAGCACTTTCATGCGACTAATGAACCATGTTTTGAGGCACTTTATTGGCAAGTTTGTCATTGTTTACTTTGATG

CRca_group_H ATGCCTTTTGGCTTGACCAATGCCCCTAGCACCTTTATGCGCCTCATGAACCATGTCTTGAGACACTTCATTGGTAAATTCGTCATTGTTTACTTTGATG

.........|.........|.........|.........|.........|.........|.........|.........|.........|.........300

CRcc_group_A ATATCTTAATTTACAGCAAGAACAAAGAGGAGCACATTAACCATCTTCAACAAGTAATGAGAGTTCTTCGTCAAAGTTCGCAGGGCATCAA---------

CRce_group_A ATATTTTAATTTATAGCAAGAACAAAGAGGAGCACATTAACCATCTTCAGCAAGTAATGCGAGTTCTTCGTCAAGAAAAGCTCTATATCAACTTGAAGAA

CRcc_group_B ATATACTGATCTTTAGCAAGTCTTTAGAAGAGCATGTTGAGCATTTGCGACTTGTTTTAAGTGCCTTGCGTGAAAATAGGCTATTTGCTAACATGGAAAA

CRce_group_B ATATATTGATTTTTAGCCAGTCTTTAGATGAACATGTTGAGCATTTGCGACTTGTTTTAAGTGCCTTGCGTGAAAATAGGTTGTTTGCTAACATGGAAAA

CRca_group_B ATATATTGATTTTTAGCCAGTCTTTAGATGAACATGTTGAGCATTTGCGACTTGTTTTAAGTGCCTTGCGTGAAAATAGGTTGTTTGCTAACATGGAAAA

CRca_group_Y ATATTCTCATTTATAGCACAAGTCTAGAGGAACATTTACAGCATGTTAAACTTGTGTTAGAAATACTTCGAAAGGAACGCCCATATGCTAATCTAAAGAA

CRcc_group_C ATATCCTAATTTATAGCAAATCTTATGATGAACACCTAGAACATATTAGGGCTGTTATGGATGTACTTCGAAGAGAAAAGCTCTATGCCAATCTCAAGAA

CRce_group_C ATATCCTAATTTATAGCAAATCTTATGATGAACACCTCGAGCATATTAGGGCTGTTATGGATGTACTTCGACGAGAGCAGCTCTATGCCAATCTCAAGAA

CRca_group_C ATATCCTAATTTATAGCAAATCTTATGATGAGCACCTAGAGCATATTAGGGCTGTTATGGATGTACTTCGAAGAGAGAAGCTCTATGCCAATCTCAAGAA

CRcc_group_E ATATCCTGATTTATAGTAGGAGCTTCGATGAGCATGTTGAACATGTGAAGCTTGTTCTTGATGTACTTCGAAGGGAAAAGCTCTATGCTAACCTTAAGAA

CRce_group_E ATATCCTGATTTATAGTAGGAGCTTAGATGAGCATGTTGAACATGTGAAGCTTGTTCTTGATGTACTTCGAAGGGAAAAGCTCTATGCTAACCTTAAGAA

CRca_group_E ATATCCTGATTTATAGTAGGAGCTTCGATGAGCATGTTGAACATGTGAAGCTTGTTCTTGATGTACTTCGAAGGGAAAAGCTCTATGCTAACCTTAAGAA

CRcc_group_D GTATTCTCATATACAGCAAGAGTCCTGAGGAACATGTAGCACATGTACGAACTCTTCTTGATGTTTTGCGTAGGGAGAGGTTATTTGCTAACCTTGGCAA

CRcc_group_F ATATTCTGATTTATAGCAGAAGCTTAGAGGAACACCTTGAGCACCTCAAAGCCATCTTTGAAGTACTTCGAAGGGAAAGGCTATATGCCAACCTTAAGAA

CRce_group_F ATATTCTGATTTATAGTAGAAGCTTAGAGGAACACTTTGAGCACCTCAAAGCCATCTTTGCAGTACTTCGAAGGGAAAGGCTATATGCCAACCTTAAGAA

CRca_group_F ATATTCTGATTTATAGCAGAAGTTTAGAAGAACACCTTGAGCACCTCAAAGCCATCTTTGAAGTACTTCGAAGGGAAAGGCTATATGCCAACCTTAAGAA

CRcc_group_G ACATATTGATCTATAGTAAGAGTACAGAAGAGCATGTTGTGCATGTACGAATGGTCTTAGATGCACTTCGAAAGGCGAGCCTCTATGCTAACCTTAAGAA

CRca_group_G ACATATTGATCTATAGTAAGAGTACAGAAGAGCATGTTGTGCATGTACGAATGGTCTTAGATGCACATCGAAAGGCGAGCCTCTATGCTAACCTTAAGAA

CRce_group_X ATATCCTCATTTATAGTAAAAGTTTAGATGAGCATGTTGATCATGTTAAAGCTGTTTTAGAGGTTCTTCGAAGGGAACACTTGTATGCTAATCTTCAAAA

CRca_group_X ATATCCTCATTTATAGTAAAAGTTTAGATGAGCATGTTGAGCATGTTAAAGCTATTTTAGAGGTTCTTCGAAGGGAACACTTGTATGCTAATCTTCAAAA

CRcc_group_H ACATCTTGATATATAGTCGTAGTGAGCAAGAGCACTTGGAGCATGTACGACTAGTTCTTGAGACGCTTCGCCAAGCACAACTCTACGCTAACCTCAAGAA

CRce_group_H ACATCCTGATCTATAGTCGTAGTGAGCATGAGCACTTAGAGCATGTGAGATTAGTTCTTGAGACACTTCGCCAGGCAAGTCTATACGCCAACCTCAAGAA

CRca_group_H ACATCTTGATATATAGTCGTAGTGAGCAAGAGCACTTGGAGCATGTACGACTAGTTCTTGAGACGCTTCGCCAAGCACAACTCTACGCTAACCTCAAGAA

.........|.........|.........|.........|.........|.........|.........|.........|.........|.........400

CRcc_group_A -------------------------------------------------------

CRce_group_A GTGTACTTTCATGGCTCCTAGTATTGTATTTTTGGG----------AGTTCGCAG

CRcc_group_B ATGTGTCTTTTGCACTTCTGAGGTTAACTTTCTTGGTTATATTGTTAGTTCGCAG

CRce_group_B ATGTGTCTTCTGCACTCCTGAAGTTAATTTCCTTGGATATATTGTTAGTTCGCAG

CRca_group_B ATGTGTCTTCTGCACTCCTGAAGTTAATTTCCTTGGATATATTGTTAGTTCGCAG

CRca_group_Y ATGTACTTTTTGCACTGACCAACTAGCGTTCCTAGGCTATGTTGTGAGTTCGCAG

CRcc_group_C GTGCAATTTTTGCACTAATGAGCTTGTGTTTCTAGGGTTTGTTATAAGTTCGCAG

CRce_group_C GTGTAATTTTTGCACTAATGAGCTTGTGTTTCTAGGGTTTGTTATAAGTTCGCAG

CRca_group_C GTGCAATTTTTGCACTAACGAACTTGTGTTCCTAGGGTTTGTTATAAGTTCGCAG

CRcc_group_E GTGCTCATTTTGTACTGATCAACTTGTCTTCCTAGGCTTTGTTGTGAGTTCGCAG

CRce_group_E GTGCTCCTTTTGTACTGATCAACTTGTCTTCCTAGGCTTTGTTGTGAGTTCGCAG

CRca_group_E GTGCTCCTTTTGTACTGATCAACTTGTCTTCCTAGGCTTTGTTGTGAGTTCGCAG

CRcc_group_D ATGTATTTTCTGCACTAATGAGCTTGTTTTCCTTGGTTATAAGGTTAGTTCGCAG

CRcc_group_F GTGCACATTTTGCACTGATCGTGTTGTGTTTCTAGGATATGTTGTAAGTTCGCAG

CRce_group_F GTGCACATTTTGCACTGATCGTGTTGTGTTTCTAGGATATGTTGTAAGTTCGCAG

CRca_group_F GTGCACATTTTGCACTGATCGTGTTGTGTTTCTAGGATATGTTGTAAGTTCGCAG

CRcc_group_G GTGTTCTTTTTGCACTAATCAACTTGTCTTCCTAGGTTATGTTGTGAGTTCGCAG

CRca_group_G GTGTTCTTTTTGCACTAATCAACTTGTCTTCCTAGGTTATGTTGTGAGTTCGCAG

CRce_group_X GTGTACCTTTTGCACTAGCGAGATTGTGTTCCTAGGATATGTTGTAAGTTCGCAG

CRca_group_X GTGTATCTTTTGCACTAATGAGGTTGTGTTCCTAGGATATGTTGTAAGTTCGCAG

CRcc_group_H ATGTACTTTTTGTACTAATGAACTTGTGTTTCTTGGCTATGTGGTAAGTTCGCAG

CRce_group_H GTGCACCTTTTGTACTAACGAGCTTGTGTTTTTAGGCTATGTGGTAAGTTCGCAG

CRca_group_H ATGTACTTTTTGTACTAATGAACTTGTGTTTCTTGGCTATGTGGTAAGTTCGCAG

.........|.........|.........|.........|.........|....455bp

Reverse ...................................GCTATGTCGTGAGTTCGCAG

Primer position

**Supplemental data 8.** Nucleotide alignment of RT domain sequences of all CRC groups and selection of PCR primers.


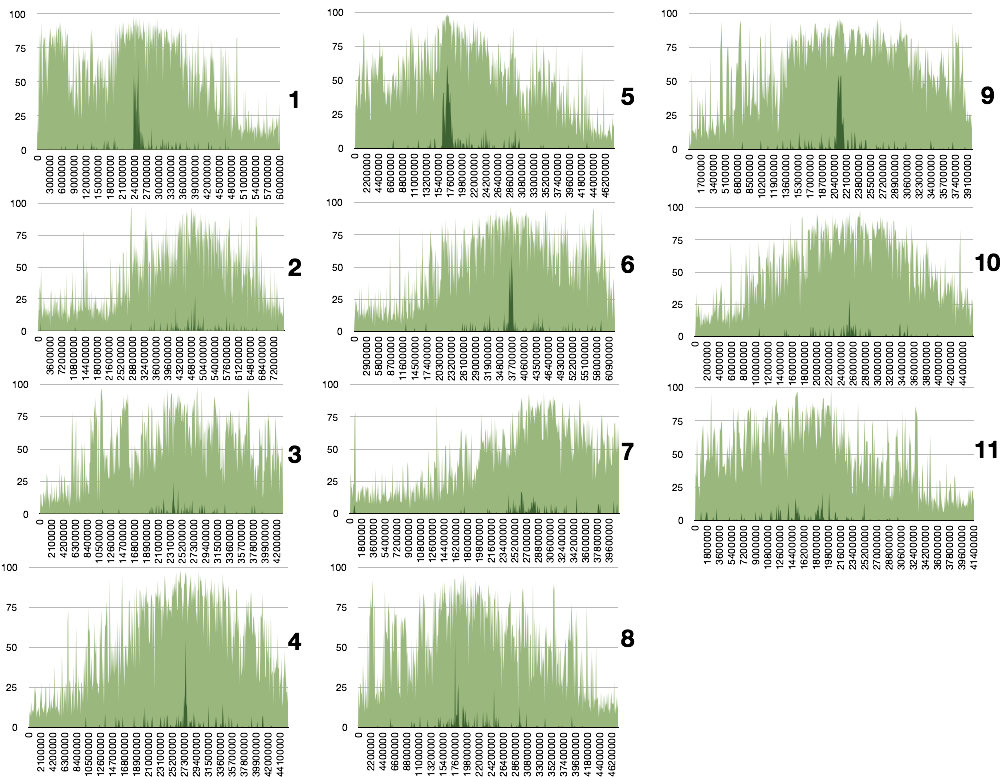


**Supplemental data 9.** The density of transposable elements (light green, annotated on *C. canephora;* Denoeud et al., 2014) and full-length CRC elements (dark green) along all *C. canephora* PacBio pseudochromosomes. Y-axis represents the density of transposable elements (A percentage calculated as the length (bp) of transposable elements over a tilling window of 100,000 bp) and X-axis the bin coordinates (every 100,000 bp) along each pseudochromosomes. Densities were calculated by DensityMap (Guizard et al., 2016).


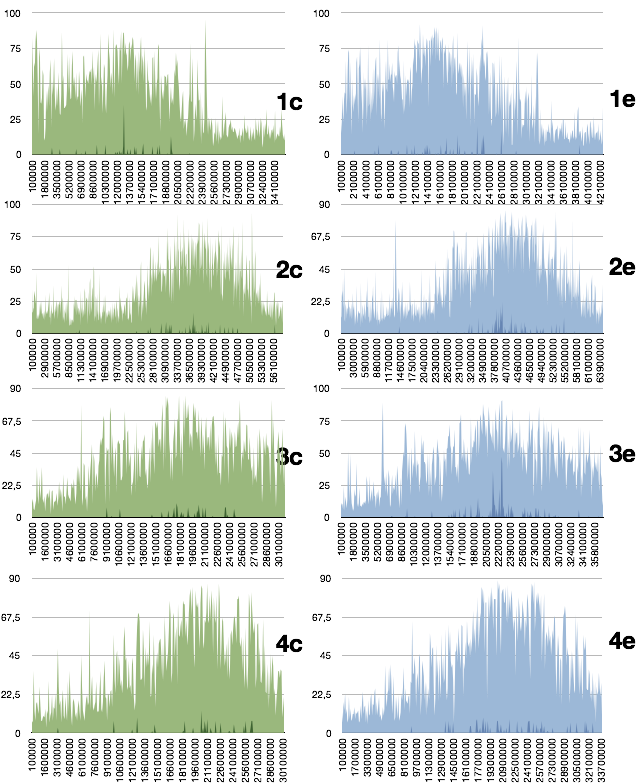


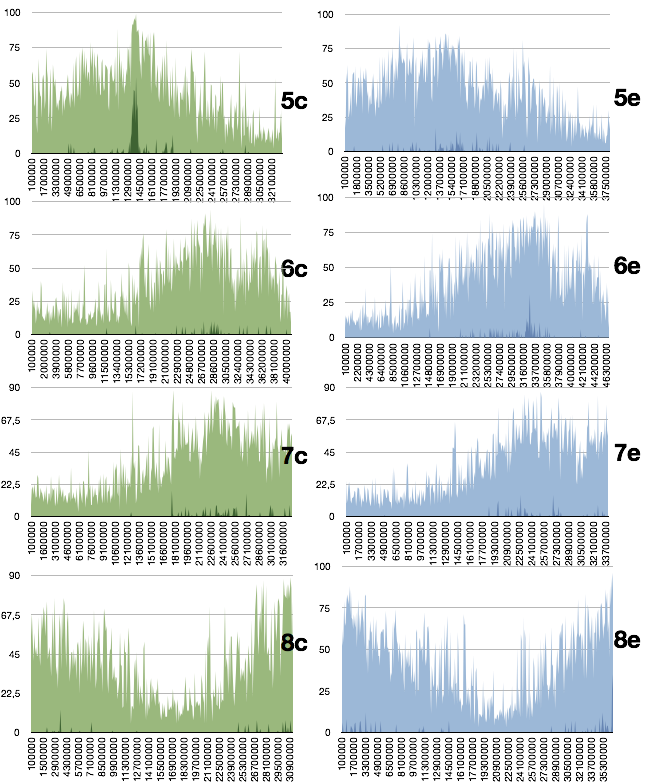


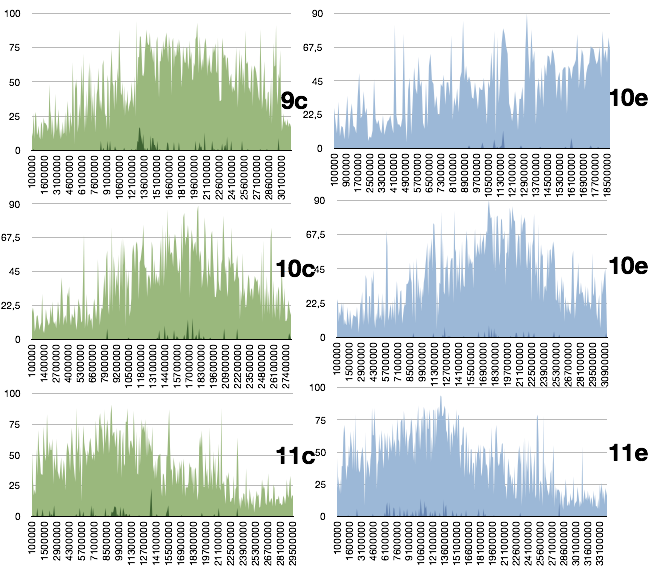


**Supplemental data 10.** The density of transposable elements (light green or light blue, annotated on *C. canephora;* Denoeud et al., 2014) and full-length CRC elements (dark green or dark blue) along all *C. arabica* PacBio pseudochromosomes. Y-axis represents the density of transposable elements (A percentage calculated as the length (bp) of transposable elements over a tilling window of 100,000 bp) and X-axis the bin coordinates (every 100,000 bp) along each pseudochromosomes. Densities were calculated by DensityMap (Guizard et al., 2016). 1c to 11c represent *C. canephora* subgenome and 1e to 11e represent *C. eugenioides* subgenomes.

*
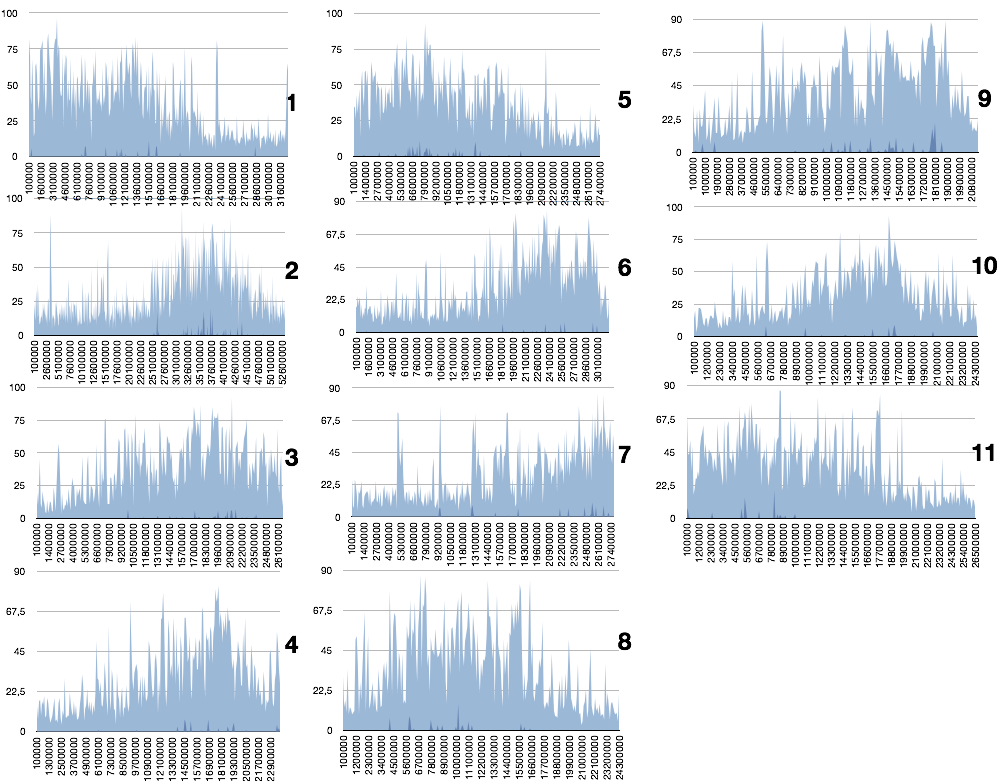
*

**Supplemental data 11.** The density of transposable elements (light blue, annotated on *C. canephora;* Denoeud et al., 2014) and full-length CRC elements (dark blue) along all *C. eugenioides* PacBio pseudochromosomes. Y-axis represents the density of transposable elements (A percentage calculated as the length (bp) of transposable elements over a tilling window of 100,000 bp) and X-axis the bin coordinates (every 100,000 bp) along each pseudochromosomes. Densities were calculated by DensityMap (Guizard et al., 2016).
